# A brain tumor computer-aided diagnosis method with automatic lesion segmentation and ensemble decision strategy

**DOI:** 10.3389/fmed.2023.1232496

**Published:** 2023-09-29

**Authors:** Liheng Yu, Zekuan Yu, Linlin Sun, Li Zhu, Daoying Geng

**Affiliations:** ^1^Academy for Engineering and Technology, Fudan University, Shanghai, China; ^2^Center for Shanghai Intelligent Imaging for Critical Brain Diseases Engineering and Technology Research, Huashan Hospital, Fudan University, Shanghai, China; ^3^Greater BayArea Institute of Precision Medicine (Guangzhou), Fudan University, Nansha District, Guangzhou, Guangdong, China; ^4^Department of Radiology, Shanghai Chest Hospital, School of Medicine, Shanghai Jiao Tong University, Shanghai, China; ^5^Department of Radiology, Huashan Hospital, Fudan University, Shanghai, China; ^6^Institute of Functional and Molecular Medical Imaging, Fudan University, Shanghai, China

**Keywords:** gliomas, brain metastases, automatic diagnosis, radiomics, ensemble

## Abstract

**Objectives:**

Gliomas and brain metastases (Mets) are the most common brain malignancies. The treatment strategy and clinical prognosis of patients are different, requiring accurate diagnosis of tumor types. However, the traditional radiomics diagnostic pipeline requires manual annotation and lacks integrated methods for segmentation and classification. To improve the diagnosis process, a gliomas and Mets computer-aided diagnosis method with automatic lesion segmentation and ensemble decision strategy on multi-center datasets was proposed.

**Methods:**

Overall, 1,022 high-grade gliomas and 775 Mets patients’ preoperative MR images were adopted in the study, including contrast-enhanced T1-weighted (T1-CE) and T2-fluid attenuated inversion recovery (T2-flair) sequences from three hospitals. Two segmentation models trained on the gliomas and Mets datasets, respectively, were used to automatically segment tumors. Multiple radiomics features were extracted after automatic segmentation. Several machine learning classifiers were used to measure the impact of feature selection methods. A weight soft voting (RSV) model and ensemble decision strategy based on prior knowledge (EDPK) were introduced in the radiomics pipeline. Accuracy, sensitivity, specificity, and the area under the receiver operating characteristic curve (AUC) were used to evaluate the classification performance.

**Results:**

The proposed pipeline improved the diagnosis of gliomas and Mets with ACC reaching 0.8950 and AUC reaching 0.9585 after automatic lesion segmentation, which was higher than those of the traditional radiomics pipeline (ACC:0.8850, AUC:0.9450).

**Conclusion:**

The proposed model accurately classified gliomas and Mets patients using MRI radiomics. The novel pipeline showed great potential in diagnosing gliomas and Mets with high generalizability and interpretability.

## 1. Introduction

Gliomas and brain metastases (Mets) are the most common brain malignancies with high cancer-related mortality (1, 2). Gliomas account for more than 50% of the overall tumors in the central nervous system (CNS) and make up 81% of total CNS malignancies with a median survival of ∼12–15 months (3–5). Mets with multiple enhancing lesions are frequent, and the incidence of Mets ranges from 10 to 40% in adult cancer patients (6, 7). The size and number of Mets determine patients’ subsequent treatment and management. Therefore, the accurate diagnosis of patients with brain tumor types will significantly impact the treatment strategy and clinical prognosis of patients.

Among many imaging techniques, magnetic resonance imaging (MRI) sequences (1) are well-suited for brain tumor diagnosis (8, 9). Contrast-enhanced T1-weighted (T1-CE) sequence and T2-fluid attenuated inversion recovery (T2-flair) sequence (10, 11) are relatively easier to acquire that can reflect tumor structural information. Radiologists read MRI images and initially determine the presence and specific location of brain tumors. However, it is time-consuming for radiologists to read too many MRI slices. Moreover, manually reading greatly depends on the radiologists’ expertise, which has interpretation bias and carries the risk of missing and misdiagnosing tumors. Therefore, an effective computer-aided diagnosis (CAD) technique with high diagnosis accuracy is much needed (12).

Radiomics is an emerging method that can extract features from the region of interest (ROI) in images of different modalities. Using machine learning algorithms, quantitatively analyzing molecular and genetic changes implicated in medical images can predict tumor type, grade, and so on. In the previous study (8, 9, 13–17), Tateishi (14)classified gliomas and Mets based on texture features from T1-CE, T2, and ADC on 127 total patients and got the best performance of 0.92 AUC. Liu (16) et al. extracted radiomics features from T1-CE of 268 patients and gave the best results in terms of accuracy (0.85) and AUC (0.93). Priya (17) et al. obtained T1, T1-CE, T2, FLAIR, and ADC from 60 patients with gliomas and 60 patients with Mets. They found that the LASSO classifier reached the best result of accuracy (0.892), AUC (0.953), sensitivity (0.887), and specificity (0.897) based on the shape, texture, and first-order features. These related works have corroborated the potential benefit of radiomics-based methods for the differentiation of gliomas and Mets.

Machine learning and optimization methods have also made progress in natural and medical image analysis (18–23). For example, in the field of brain tumor diagnosis, Pugalenthi (18) et al. enhanced the tumor section based on Social Group Optimization (SGO) algorithm-assisted Fuzzy–Tsallis thresholding. Rinesh (19) et al. proposed the combination of k-based clustering processes to locate the tumor in hyperspectral imaging. The value of k is determined using the firefly algorithm. The optimization processes reduced the manual calculation for finding K’s optimal value to segment the brain regions. Gopal (20) et al. proposed a majority voting-based ensemble algorithm to optimize the overall performance of brain tumor grading. Ahmadi (21) et al. performed a PSO algorithm to optimize the gradient descent algorithm during brain tumor classifier training.

However, these previous works (8, 9, 13–17) used manual or semiautomatic methods for ROI segmentation, which was labor-intensive and possessed potential bias. Furthermore, they were based on a single center usually lacking external validation, and the obtained data were not large enough. Finally, some advanced MRI sequences or other functional sequences (24) had poor clinical applicability in some primary hospitals.

Our main contributions are summarized as follows. First, an integrated gliomas and Mets CAD pipeline was proposed, including two improved segmentation models and the radiomics-based classifier, which could reduce the cost of manual annotation in the traditional radiomics pipeline. Second, an ensemble decision strategy based on prior knowledge (EDPK) was introduced in the pipeline to reduce the impact of automatic segmentation uncertainty on final classification performance and improve diagnostic accuracy. Finally, the more accessible T1-CE and T2-flair sequences were obtained from three hospitals in the study, which were reliable, clinically applicable, and could help radiologists in the diagnostic process.

The remaining sections of the article are structured as follows: A detailed description of all the materials and methods including MRI acquisition, feature extraction, feature selection, model construction, and our EDPK strategy is presented in Section 2. The results of brain tumor classification and verification procedures are presented in Section 3. The discussion is provided in Section 4. Finally, the conclusion is added in Section 5.

## 2. Materials and methods

**Figure 1:**
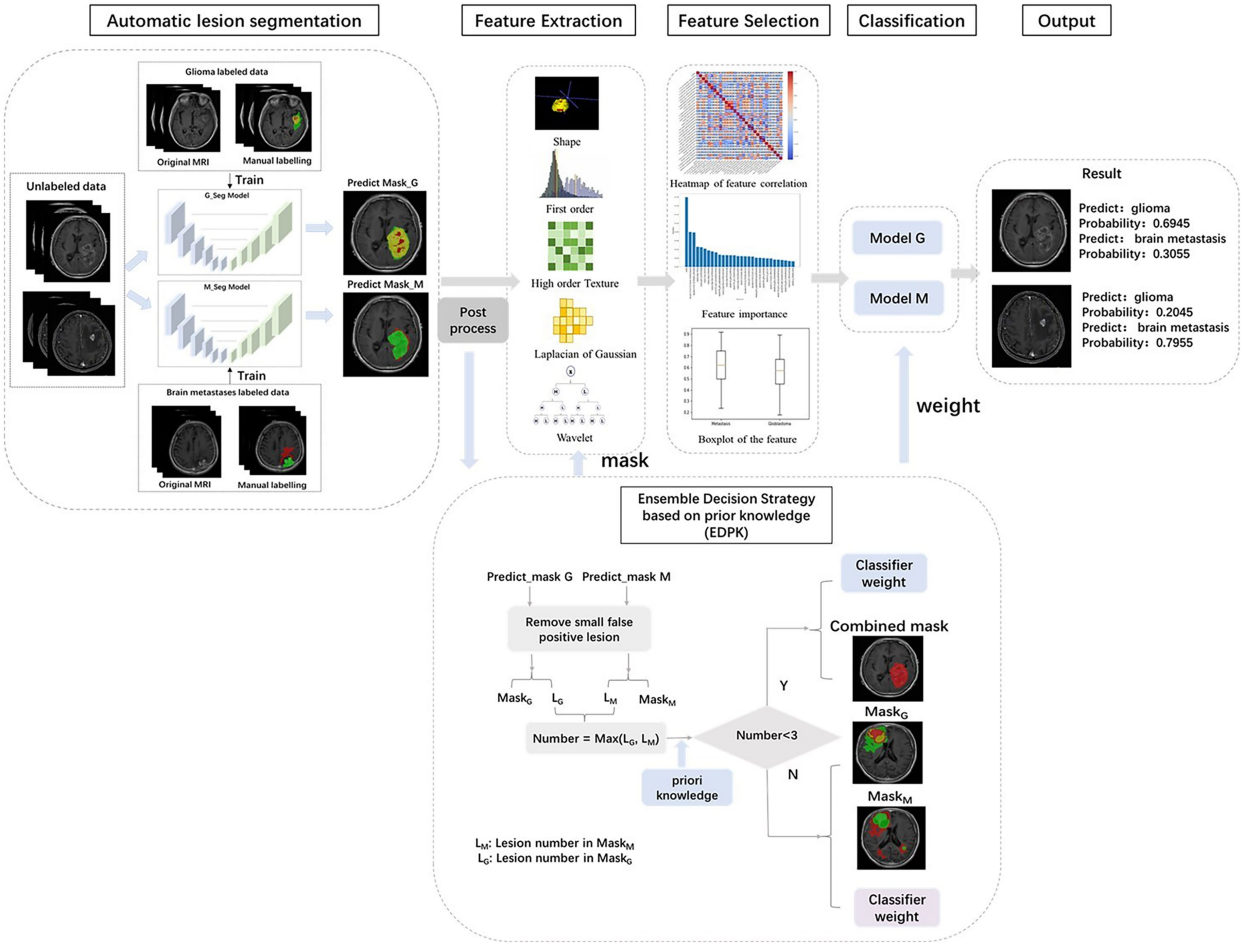
Workflow of our study. MRI images of 1797 brain tumor patients were collected from three hospitals. Lesion segmentations were performed automatically using deep learning model. The ensemble decision strategy based on prior knowledge was included in the radiomics pipeline.

Figure 1 summarizes the different steps adopted in this study.

### 2.1. MRI acquisition

The present study included a total of 1,022 gliomas and 775 Mets patients from three hospitals under approval by the institutional review boards (IRBs). The enrolled patients met the inclusion criteria: (a) diagnosis of high-grade gliomas or Mets and (b) all MR scans were performed before initiating treatment. The exclusion criteria were as follows: (a) image artifacts or incomplete images and (b) a history of other CNS diseases.

These MR images were preoperatively scanned with SimensVerio3.0 T, GE750W3.0 T and obtained complying with clinical criteria and protocol. All images were in Neuroimaging Informatics Technology Initiative (NIfTI) format after the data were desensitized. The protocol included the T1-CE and T2-flair sequence. According to the same annotation protocol (annotated by two resident radiologists, reviewed by one attending radiologist), experienced radiologists used open-source ITK-SNAP (version 3.2, http://www.itksnap.org/pmwiki/pmwiki.php) (25) software to delineate ROI including the edema, the necrotic, and the enhancing tumor area. The whole ROI was the combination of these regions and was merged for subsequent feature extraction.

### 2.2. Image preprocessing

Image preprocessing was performed to standardize images. First, the N4 bias field correction (26) was performed to reduce the low-frequency intensity non-uniformity. All T1-CE sequences acquired during the same session were registered to the T2-flair sequence using ANTsPy^1^ package (27). Then to ensure the physical space consistency of each voxel in different images, all images were resampled to 1 × 1 × 1 mm^3^ voxel size using the Simple Insight Segmentation and Registration Toolkit (SimpleITK, https://github.com/SimpleITK/SimpleITK) package (28). To reduce the effect of differences in image intensity, intensity normalization was applied to all MRI images with the Z-score normalization method (29) using the mean and standard deviation for the entire brain area.

### 2.3. Tumor region of interest segmentation

**Figure 2:**
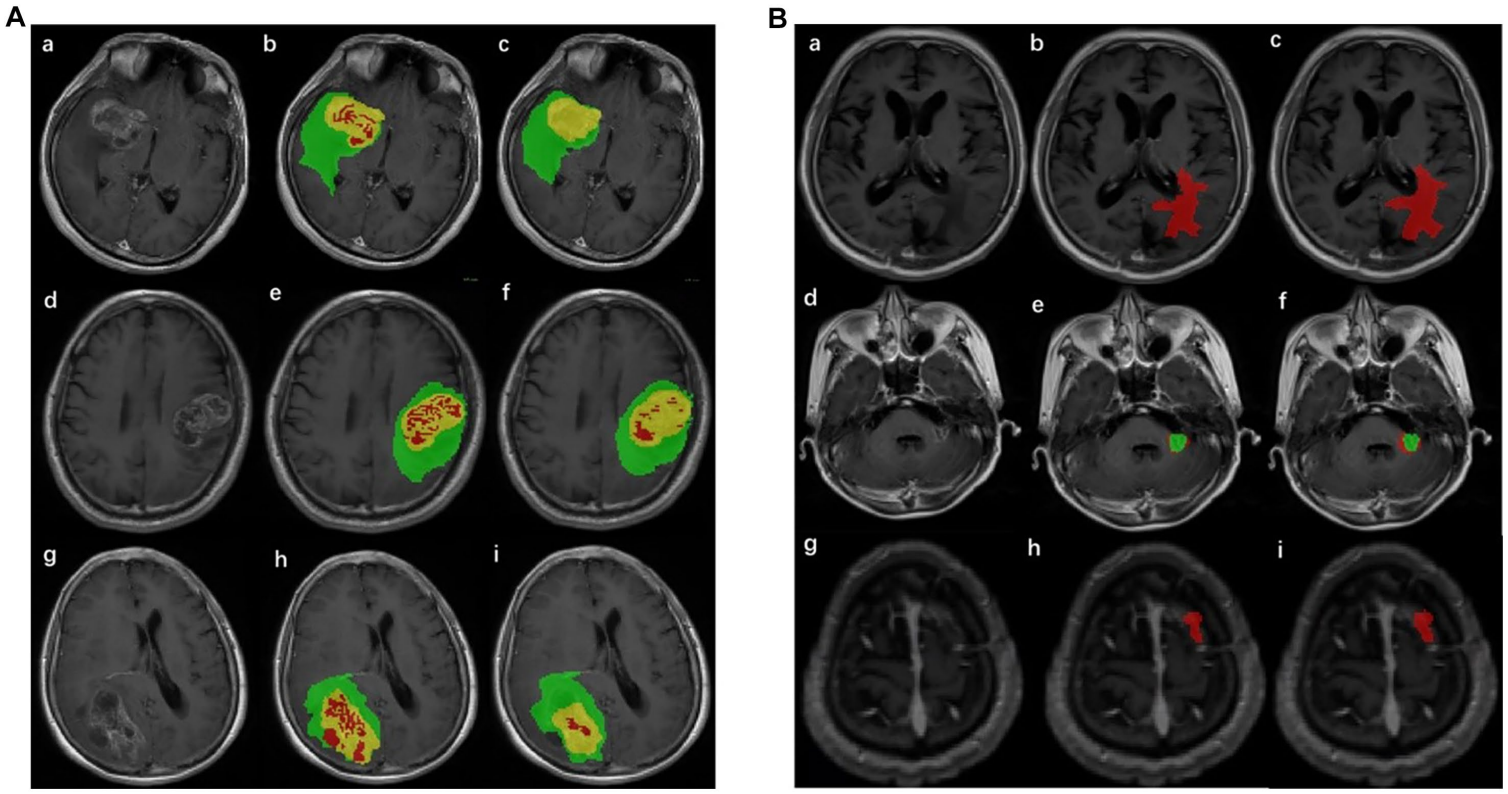
Result of lesion automatic segmentation. **(A)** Result of gliomas: green label indicates enhancing tumor, yellow indicates peritumoral edema, and red indicates necrotic tumor area. (a), (d), and (g) were original MRIs; (b), (e), and (h) were ground truth; (c), (f), and (i) were the results of the segmentation model of gliomas. **(B)** Result of Mets: red label indicates peritumoral edema and green indicates enhancing tumor. (a), (d), and (g) were original MRIs; (b), (e), and (h) were ground truth; (c), (f), and (i) were the results of the segmentation model of Mets.

The annotation of the tumor ROI is the preparation for tumor classification. It was difficult to segment glioma and Mets lesions by only one segmentation model; thus, two segmentation models were implemented in the pipeline. The structure of the improved segmentation model is detailed in Supplementary Figure S1. Two models were based on revised U-net (30) architecture, incorporating the DenseNet (31) and self-attention (32) and used T1-CE and T2-flair images with sizes of 160 × 160 × 16 as network inputs. The glioma segmentation model yielded three regions including enhancing tumor, necrotic tumor, and peritumoral edema. The Mets segmentation model yielded two regions including enhancing tumor and peritumoral edema. These regions were compatibly applied to both MRI sequences (T1-CE and T2-flair). The whole ROI was the combination of these regions and was merged for subsequent quantitative feature extraction. The result of the automatic lesion segmentation is shown in Figure 2.

### 2.4. Radiomics feature extraction

Features were automatically extracted using the Pyradiomics package (33) from the whole ROI. Two filters, Wavelet transform and Laplacian of Gaussian (LoG) with two sigma levels (3.0 and 5.0), were used during the feature extraction. Three kinds of images were used to extract these features: shape features, first-order features, or high-order texture features. Shape features described the three-dimensional size and shape of the ROI, which were independent of the gray-level intensity distribution. First-order features described the distribution of voxel intensities within the ROI. High-order texture features can reflect heterogeneity within a lesion and be extracted by using the gray-level co-occurrence matrix (GLCM), gray-level dependence matrix (GLDM), gray-level run length matrix (GLRLM), and gray-level size zone matrix (GLSZM).

A total of 960 features from each sequence, which included 14 shape features, 198 first-order features (18 were from original images, 36 were from LoG images, 144 were from wavelet images), and 748 high-order features (68 were from original images, 136 were from LoG images, 544 were from wavelet images), were obtained.

### 2.5. Feature preprocessing

Before feature selection, all extracted features were standardized using the Z-score normalization method, uniformly converting the magnitudes of different features into the same magnitude to ensure consistency. For each feature vector, the mean and standard deviation were calculated (in training sets) and then normalized using Z-score normalization, which consists of subtracting each feature vector from the mean followed by division by the standard deviation. For Z-score normalization, the mean and standard deviation were calculated for the training set and then applied to the testing set. Features’ correlation was evaluated using Pearson’s correlation.

### 2.6. Feature selection and classification

In this study, the Mann–Whitney U-test was implemented to choose features with statistical differences (value of *p* <0.05) and remove the feature with the worst univariate predictive power. Then, several different combinations were tested by cross-combination of three feature selectors and six classifiers. The Least Absolute Shrinkage and Selection (LASSO) (34), Mutual Information (MI) (35), and Recursive Feature Elimination Random Forest (RFE-RF) (36) were investigated for feature selection and reduction based on the radiologist-delineated ROI. Feature preprocessing and selection were performed on training sets and then applied to testing sets. LASSO uses the L1 norm as a penalty term to change the unimportant regression coefficients to zero to eliminate variables. MI was calculated between the radiomics feature and its category. Then, the features were sorted according to MI and the top *n* features were selected according to validation performance. RFE-RF calculates the feature importance of sub-radiomics features set recursively. The Gini index was used to measure the contribution of features based on the contribution of each feature on each tree in the Random Forest. Features with high contribution were selected.

The selected features were used as the input of several ML classifiers, including Gaussian Naïve Bayes (GNB) (37), Extreme Gradient Tree (XGBoost) (38), logistic regression (LR) (39), Random Forest (RF) (40), and Support Vector Machine (SVM) (41) with two different kernels: polynomial kernel (SVM-Poly) and radial basis function (SVM-RBF). Classification algorithms were optimized during the training process using the Grid Search method. The best models were chosen by one standard deviation rule in 10-fold cross-validation and then evaluated on the test or external validation sets. Hyperparameter values of each best model used in the classification task are shown in Supplementary Table S4.

After that, the important radiomics feature sets and classifier combinations were obtained. The Pearson coefficient (42) was used to measure the correlation between selected features. The feature correlation analysis was visualized by the heat map of feature correlation coefficients. Moreover, the feature importance was visualized by the bar chart and SHAP model. We implemented these feature selection methods and classification algorithms using the SciPy library^2^ and Scikit-learn Machine Learning library (43).^3^

### 2.7. Ensemble decision strategy based on prior knowledge

In the EDPK strategy, a weight soft voting model (RSV model) was developed by combining the two best-performing classifiers based on the classification performance and using the Grid Search method to search optimal hyperparameters.

Two preprocessed MRI sequences of each patient were put into the glioma and Mets segmentation models, respectively, and two segmentation results were obtained with post-processing. EDPK strategy was made according to the lesion number in the two segmentation results (44), which could adaptively determine the tumor ROI and the weight in the RSV model. Figure 1 depicts the proposed EDPK strategy, which is also explained in Algorithm 1. Based on prior knowledge, the set number was set to 3 in this study.

**Table:** 

**Algorithm 1** Ensemble decision strategy based on prior knowledge (EDPK)
**Inpu**t: Automatic segmentation results**Predict_mask G** and**Predict_mask M** **Output:** Post-processing results:**Mask**_**G**_ and**Mask**_**M**_ The lesion number in the Mask_G_:**L**_**G**_ The lesion number in the Mask_M_:**L**_**M**_ Final mask set Classifier weight $\omega =\left\{\omega_{1},\omega_{2}\right\}$
1: Remove small false positive lesions in Predict_mask G and Predict_mask M and obtain post-processing results Mask_G_ and Mask_M_
2: Calculate the lesion number L_G_, L_M_ of Mask_G_, and Mask_M_, respectively
3: Number = Max (L_G_, L_M_)
4: if Number < set number:
5: $\omega_{1}=\omega_{2}$ Combine Mask_G_ and Mask_M_ into Mask Final mask set = {Mask}
6: else:
7: Adaptively adjust weights Final mask set = {Mask_G_, Mask_M_}
8:**Return** Final mask set and Classifier weight

### 2.8. SHAP analysis

In traditional feature importance analysis, important features can be seen, but the influence of features on prediction results cannot be seen. Shapley Additive Explanations (SHAP) (45) can reflect the influence of the features predicted by each sample through SHAP value and show the positive and negative influence.

For each predicted sample, the model generates a predicted value, and the SHAP value is the value assigned to each feature in the sample.


$$y_{i}=y_{base}+f\left(x_{i1}\right)+f\left(x_{i2}\right)+\ldots +f\left(x_{ik}\right)$$

$f\left(x_{ij}\right)$ is the SHAP value of $x_{ij}$ , which is the contribution of the jth feature in the ith sample to the final predicted value. When $f\left(x_{ij}\right)>0$ , it indicates that the feature improves the predicted value and has a positive effect. Otherwise, it indicates that this feature reduces the predicted value and has a negative effect.

## 3. Results

### 3.1. Dataset

Overall, 1,797 patients were included in this study and randomly divided into training (*n* = 1,278), validation (*n* = 319), and testing sets (*n* = 200). The details of patients’ distribution are shown in Supplementary Table S1. The training set from gliomas was used to train the glioma segmentation model, and the training set from Mets was used to train the Mets segmentation model. The validation set was used for tuning the model parameters during the training process. The testing set was used for the final model performance evaluation.

### 3.2. Performance with the addition of the clinical feature

Based on the radiologists-delineated ROI, 960 radiomics features (14 shape features, 198 first-order features, and 748 high-order features) were extracted. The number of features extracted from different image types is shown in Supplementary Table S3, and the details of the extracted features are summarized in Supplementary Table S2. First, 873 features were coarsely selected by the Mann–Whitney U-test and further filtered using LASSO.

According to clinical experiences, the lesion number helped distinguish gliomas and Mets. To verify it, the experiment was conducted using a Random Forest classifier. The result is shown in Table 1. The incorporation of the lesion number resulted in some improvement in the model classification performance. Therefore, the lesion number is added to the feature set in subsequent comparison experiments.

**Table 1:** Results of the RF classifier before and after incorporating the lesion number into the feature set.

Features	Label	ACC	AUC	SEN	SPE
57 features	0	0.8834	0.9571	0.8834	0.9044
	1	0.9044	0.9571	0.9044	0.8834
57 features, lesion number	0	**0.8912**	**0.9705**	**0.8860**	**0.9387**
	1	**0.9069**	**0.9705**	**0.9387**	**0.8860**

Label 0 represents brain metastases; Label 1 represents gliomas.

The bold values indicate the highest results.

### 3.3. Comparison of the classification performance of different combinations

Supplementary Figure S2 shows the correlation of radiomics features after LASSO feature selection in the training set, and Supplementary Figures S3, S4 show the correlation of radiomics features after MI and RFE-RF feature selection. In the heat map, a lower color saturation indicated a lower correlation between features. The heat map showed that the correlations between feature pairs were reduced after applying the LASSO selection.

**Figure 3:**
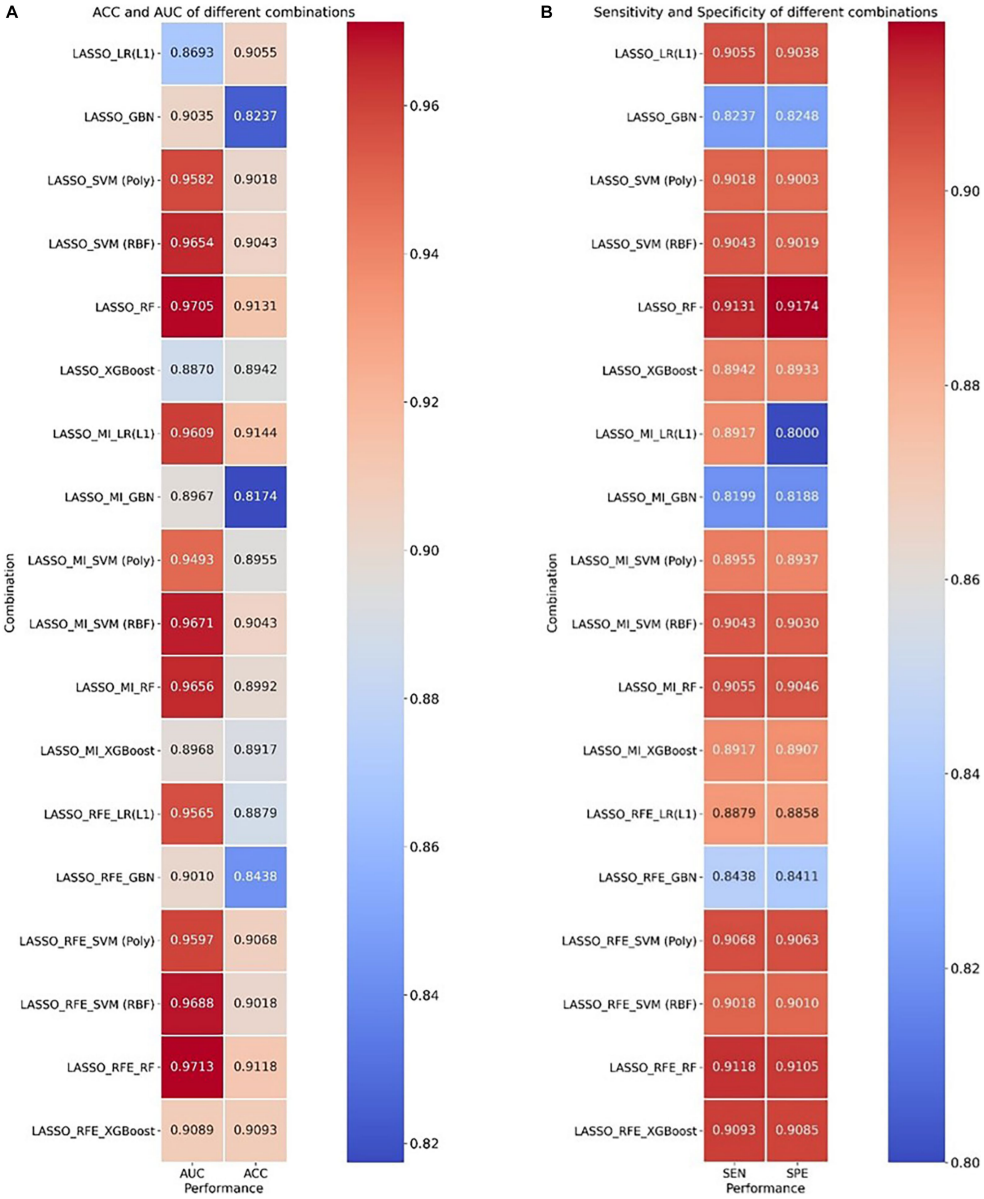
Heatmap of ACC, AUC, sensitivity, and specificity for cross-combination of feature selections and different classifiers. **(A)** ACC and AUC and **(B)** sensitivity and specificity.

Figure 3 provides the classification indices of (a) ACC and AUC and (b) SEN and SPE for different combinations, respectively, in the testing set. More detailed results are presented in Supplementary Tables S5–S9 for different combinations. The better-performing model for features selected by LASSO and MI was SVM using the RBF kernel function, with an average AUC of 0.9671 and an accuracy of 0.9043. The best-performing model for features selected by LASSO and RFE-RF was Random Forest, with an average AUC of 0.9713 and an accuracy of 0.9118. However, the combination of the two feature selection methods slightly reduced the classification performance of the classifier compared to features filtered using only LASSO, and the best-performing model for 2-class brain tumor classification was RF, with an average AUC of up to 0.9705 and an accuracy of up to 0.9131 based on the radiomics features selected by LASSO.

### 3.4. Classification performance of our model

To combine the advantages of classifiers with good classification performance, an RSV model was formed and a grid search method was used to determine the parameters of each model. Several radiomics features selected by LASSO and the lesion number were used to test the classification performance. Table 2 shows the classification performance of the RSV model with different weight settings and other classifiers, in which all evaluation metrics of the RSV model were higher than those when using the SVM model alone, indicating that the RSV model can improve the classification performance of the 2-class brain tumor.

**Table 2:** Classification performance of different classifiers on 2-class brain tumors in the testing set.

Features	Model	ACC	AUC	SEN	SPE
57 features + NL	LR(L1)	0.9055	0.8693	0.9055 (0.8756, 0.9338)	0.9038 (0.9338, 0.8756)
	GNB	0.8237	0.9035	0.8237 (0.8446, 0.8039)	0.8248 (0.8039, 0.8446)
	SVM (poly)	0.9018	0.9582	0.9018 (0.8756, 0.9265)	0.9003 (0.9265, 0.8756)
	SVM (RBF)	0.9043	0.9654	0.9043 (0.8601, 0.9461)	0.9019 (0.9461, 0.8601)
	RF	0.9131	0.9705	0.9131 (0.8860, 0.9387)	0.9174 (0.9387, 0.8860
	XGBoost	0.8942	0.8870	0.8942 (0.8782, 0.9093)	0.8933 (0.9093, 0.8782)
	RSV (1:1)	0.9144	0.9730	0.9144 (0.8990, 0.9289)	0.9135 (0.9289, 0.8990)
	**RSV (2:1)**	**0.9144**	**0.9736**	**0.9144** (0.8938, 0.9338)	**0.9132** (0.9338, 0.8938)
	RSV (2:3)	0.9068	0.9709	0.9068 (0.8938, 0.9191)	0.9061 (0.9191, 0.8938)
	RSV (4:3)	0.9144	0.9731	0.9144 (0.8964, 0.9314)	0.8995 (0.9314, 0.8964)

The bold values indicate the highest results.

**Figure 4:**
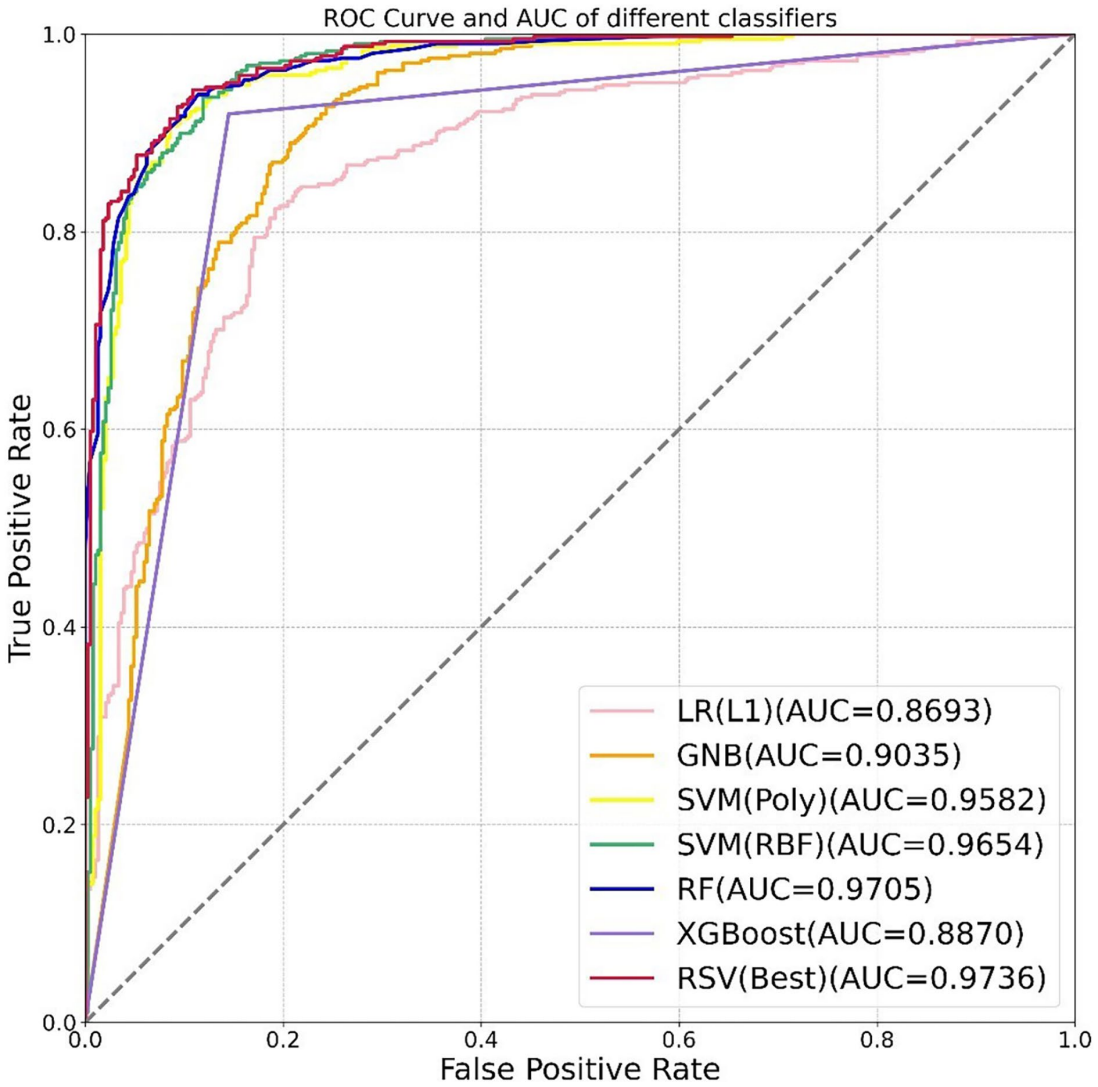
ROC curve and AUC of different models based on 58 features (lesion number + 57 radiomics features). The different colored lines represented the classification performance of the model in the testing set.

The ROC curve and AUC of different classifiers based on LASSO feature selection in the testing set are shown in Figure 4. Our RSV model (the red line) was the closest to the upper left corner, with a slightly higher sensitivity and lower false positive rate than other models. All evaluation metrics of our RSV model were higher than other classifiers, and the best classification performance the RSV model achieved was with an ACC reaching 0.9144 and AUC reaching 0.9736. ROC curve and AUC of different models based on other combinations of feature selection and classifiers are shown in Supplementary Figures S8, S9.

**Figure 5:**
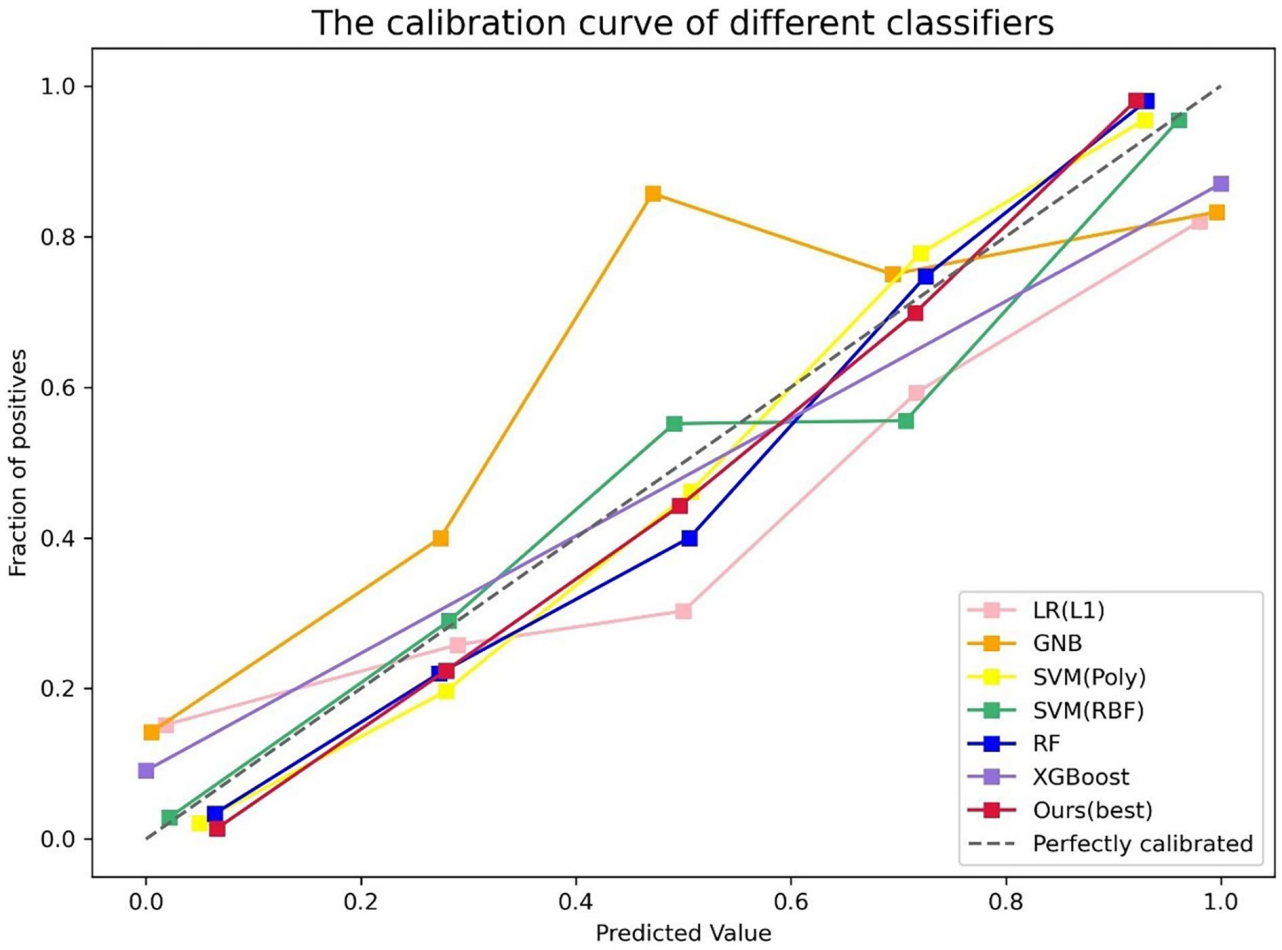
Calibration plot of different classifiers. The y-axis is fraction of positive, and the x-axis is predicted value. The different colored lines indicate the calibration curves for the different models. The dashed line is the reference line that a classifier would be like. Our model (the red line) is closer to the diagonal dashed line.

The performance of the classifier can further be quantified in terms of a calibration plot. The calibration curve of the classifier is shown in Figure 5. The dashed line is the reference of perfect calibration; thus, the closer the calibration curve of the model is to the diagonal dashed line, the more accurate the model’s predictive diagnosis is. It can be seen that the RSV model (the red line) is closer to the diagonal dashed line illustrating better predictive performance.

### 3.5. The performance of the proposed EDPK strategy

To validate the effectiveness of the EDPK strategy proposed in the study, radiomics features were extracted from automatic segmentation results; features with excellent performance were selected, and the proposed RSV model was used as the classifier. The classification performance under different segmentation precision is shown in Table 3. Compared with the results of other state-of-the-art segmentation models, two automatic segmentation models had higher dice coefficients on the testing data of gliomas and Mets, which proved the effectiveness of the two segmentation models. In addition, it could be seen that the RSV model added to the EDPK strategy had achieved the highest classification accuracy, AUC, sensitivity, and specificity. Through the EDPK strategy, the classification performance of the weighted soft voting ensemble model proposed in the study was further improved.

**Table 3:** Classification performance under different segmentation precision in the testing set.

Segmentation precision	Classifier	ACC	AUC	SEN	SPE
Dice_G = 0.8286 Dice_M = 0.6588 (Swin unetr (46))	RSV model	0.8500	0.9269	0.8500	0.8500
	RSV model with EDPK strategy	0.8700	0.9528	0.8700	0.8700
Dice_G = 0.8451, Dice_M = 0.8463**(Our two models)**	RSV model	0.8850	0.9450	0.8850	0.8850
	RSV model with EDPK strategy	**0.8950**	**0.9585**	**0.8961**	**0.8961**
Dice_G = 1, Dice_M = 1 (Radiologists delineated)	RSV model	0.9144	0.9736	0.9144	0.9132

Dice_G: the whole tumor dice in the glioma segmentation model.

Dice_M: the whole tumor dice in the brain metastases segmentation model.

Dice_G = 1, Dice_M = 1 means the whole ROI delineated by radiologists.

The bold values indicate the highest results.

The classification performance of the RSV model with different weights is given in Table 4. It can be seen that the introduction of the EDPK strategy in RSV can achieve relatively high values in terms of accuracy, AUC, sensitivity, and specificity, indicating that the EDPK strategy was effective and interpretable through the results of automatic segmentation for different decision processing.

**Table 4:** Classification performance of the RSV model with different weights based on the automatic segmentation result.

Weight in RSV model	ACC	AUC	SEN	SPE
1:1	0.8600	0.9335	0.8600	0.8600
2:1	0.8850	0.9450	0.8850	0.8850
3:1	0.8600	0.9427	0.8600	0.8600
EDPK strategy	**0.8950**	**0.9585**	**0.8961**	**0.8961**

The bold values indicate the highest results.

### 3.6. Feature importance analysis and SHAP explanation

The importance of radiomics features selected by LASSO is visualized using a bar chart in Supplementary Figure S5. A larger value in the bar chart indicated greater importance in diagnosing gliomas and Mets. It could be seen that “shape-Flatness,” “shape-Maximum3D-Diameter,” and “shape-Sphericity” were important radiomics features from the original image. The Mutual Information of features selected after MI and the importance of features selected after RFE-RF are shown in Supplementary Figures S6, S7.

**Figure 6:**
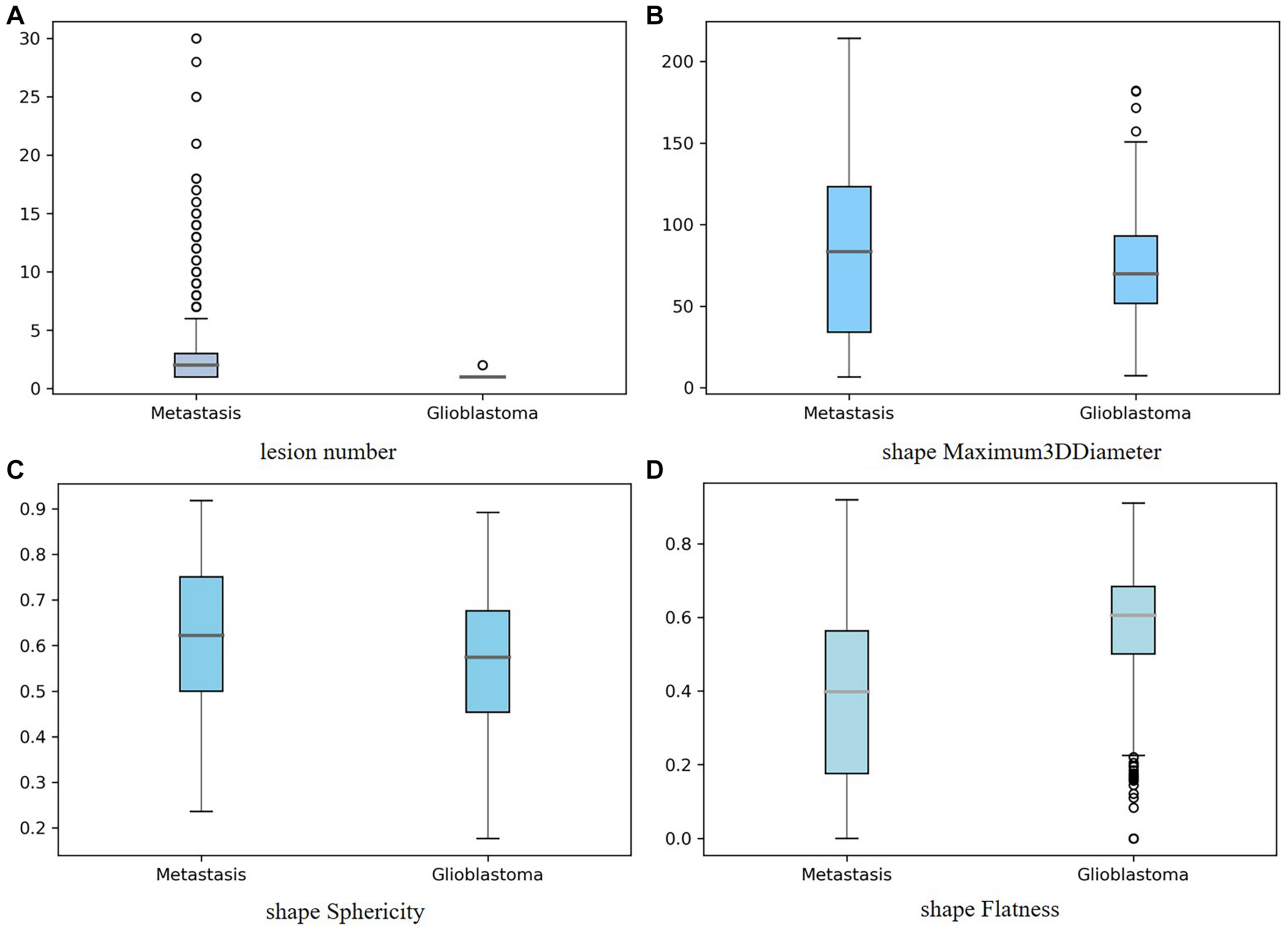
Boxplot showing the distribution of the important feature for two tumor types: **(A)** lesion number, **(B)** shape-Maximum3D-Diameter, **(C)** shape-sphericity, and **(D)** shape-Flatness. The y-axis represented the box plots of the values of the feature, while the x-axis represented two tumor types.

In Figure 6, the distribution of four important features: lesion number, shape-Maximum3D-Diameter, shape-sphericity, and shape-Flatness in two tumor types is shown in a boxplot, respectively. It could be seen that gliomas and Mets were roughly normally distributed in these features and there were fewer outliers in the distribution of these four features.

**Figure 7:**
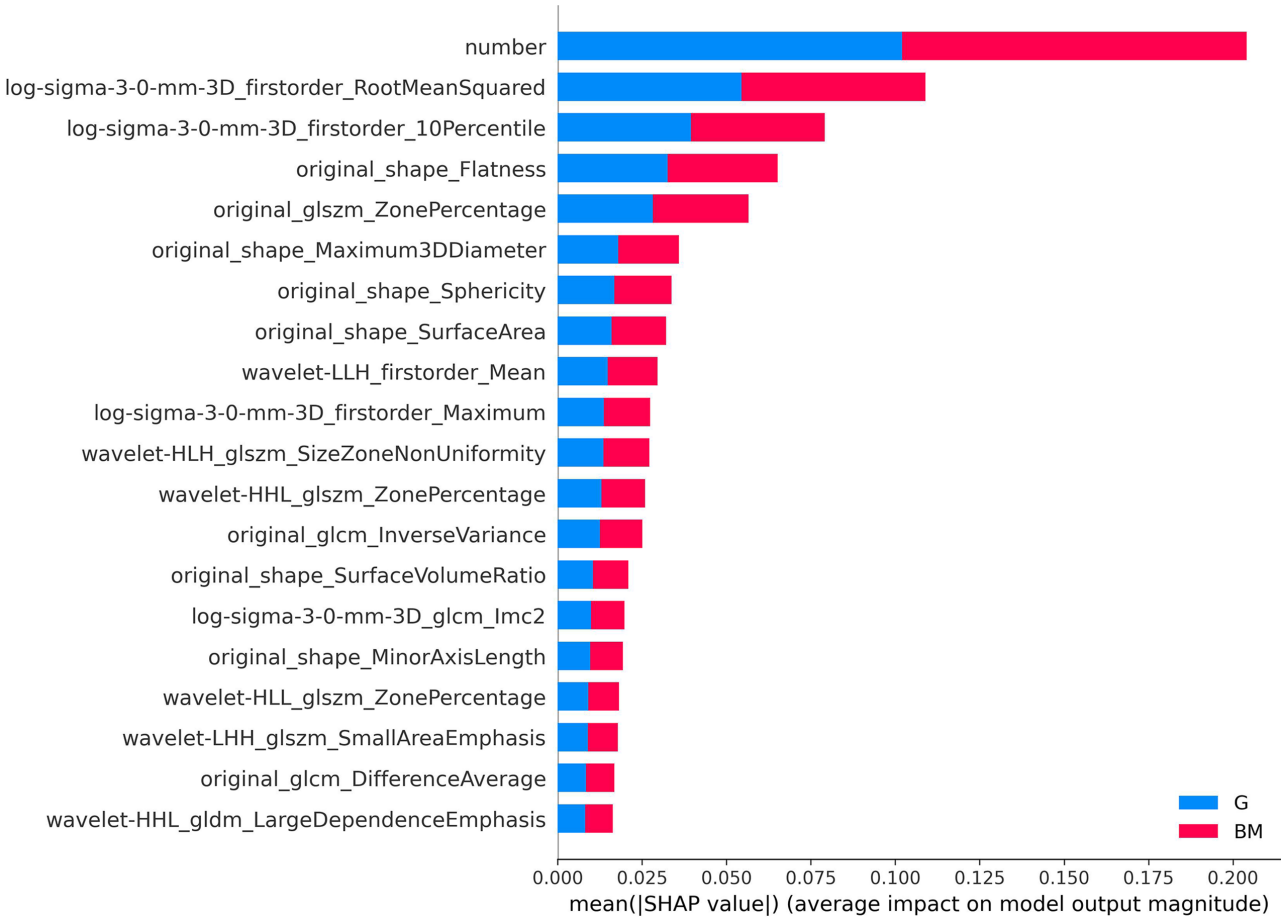
Feature importance-based SHAP in the brain tumor classification. The color represents the tumor type (red: brain metastases, blue: glioma).

The SHAP explainable model took the absolute value of the SHAP value of each feature as the importance of the feature. Figure 7 shows the importance of the top 20 features in the process of tumor type prediction. The vertical location showed the feature’s importance. It could be seen that the lesion number had the highest feature importance.

**Figure 8:**
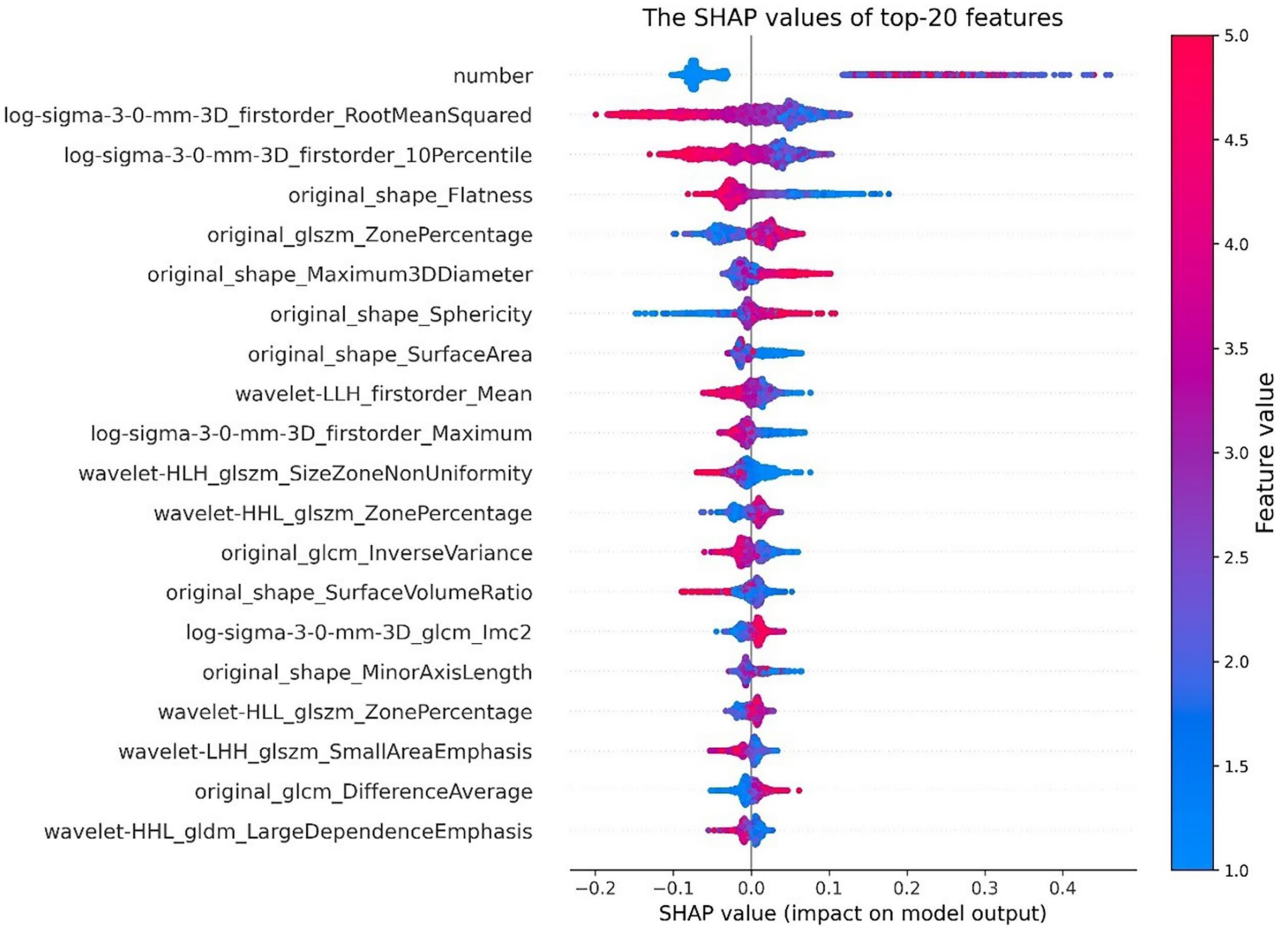
SHAP value of top 20 features. The color represents the feature value (red high, blue low).

In Figure 8, the SHAP values of the top 20 important features are plotted for each sample. The y-axis represents the features arranged by importance, and the x-axis represents the SHAP value of each feature. Each point represents a sample, and the sample size is stacked vertically. The figure shows important features and the influence range of these features on all samples.

**Figure 9:**
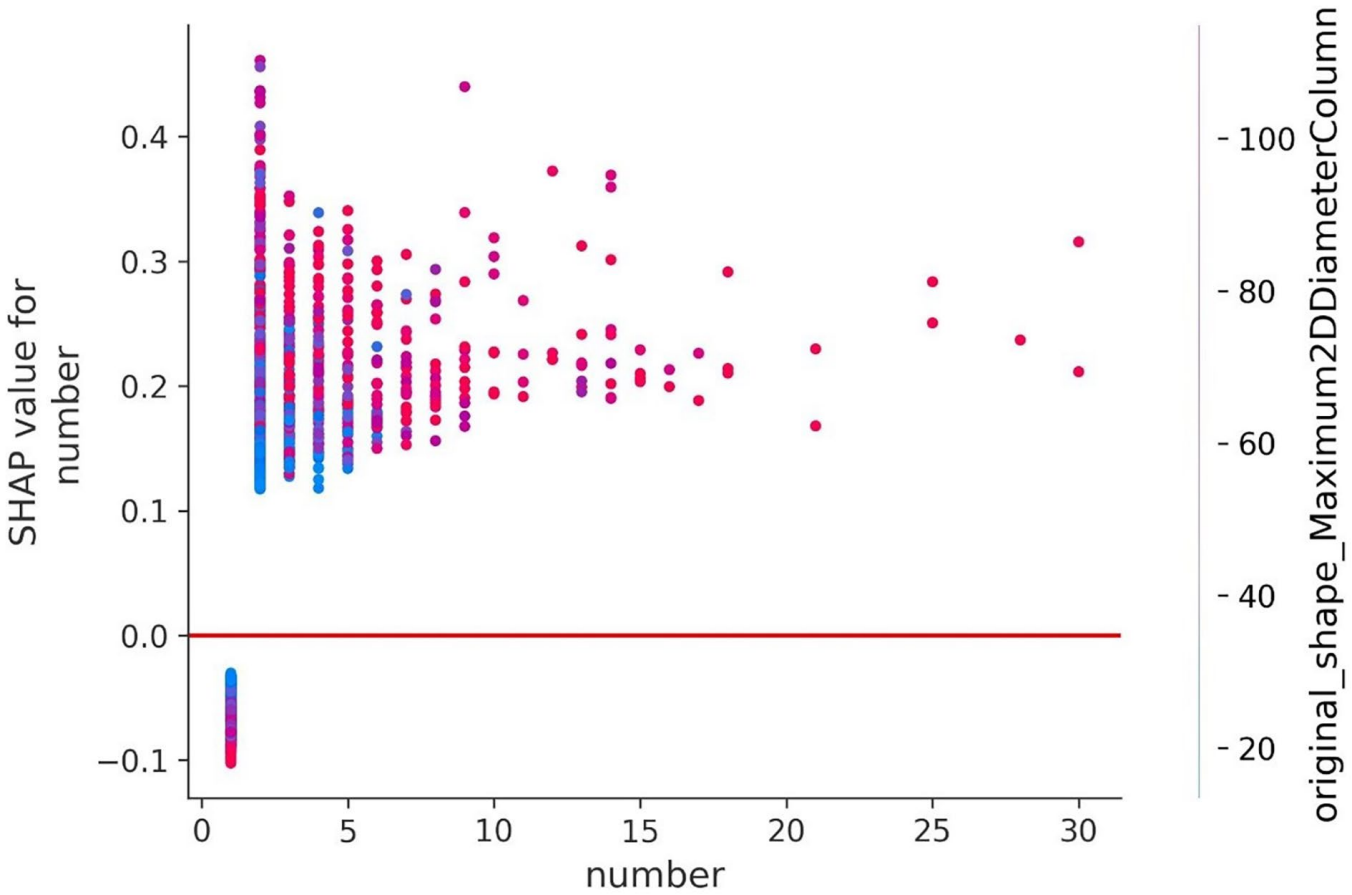
Feature dependence of the lesion number. The y-axis represents the SHAP value of the number.

To understand how a single feature affected the output of the model, taking the lesion number as an example, the SHAP value of this feature was compared with the SHAP value of the feature of all samples in the data set, and the results are shown in Figure 9. Each point represents a sample, the x-axis is the lesion number, and the y-axis is the SHAP value of the feature. It could be seen that “Original_Shape_Maximum2DDiameterColumn” is the characteristic variable that interacts with the number of lesions. The fluctuations in the vertical direction indicate the interactions between the two features.

**Figure 10:**
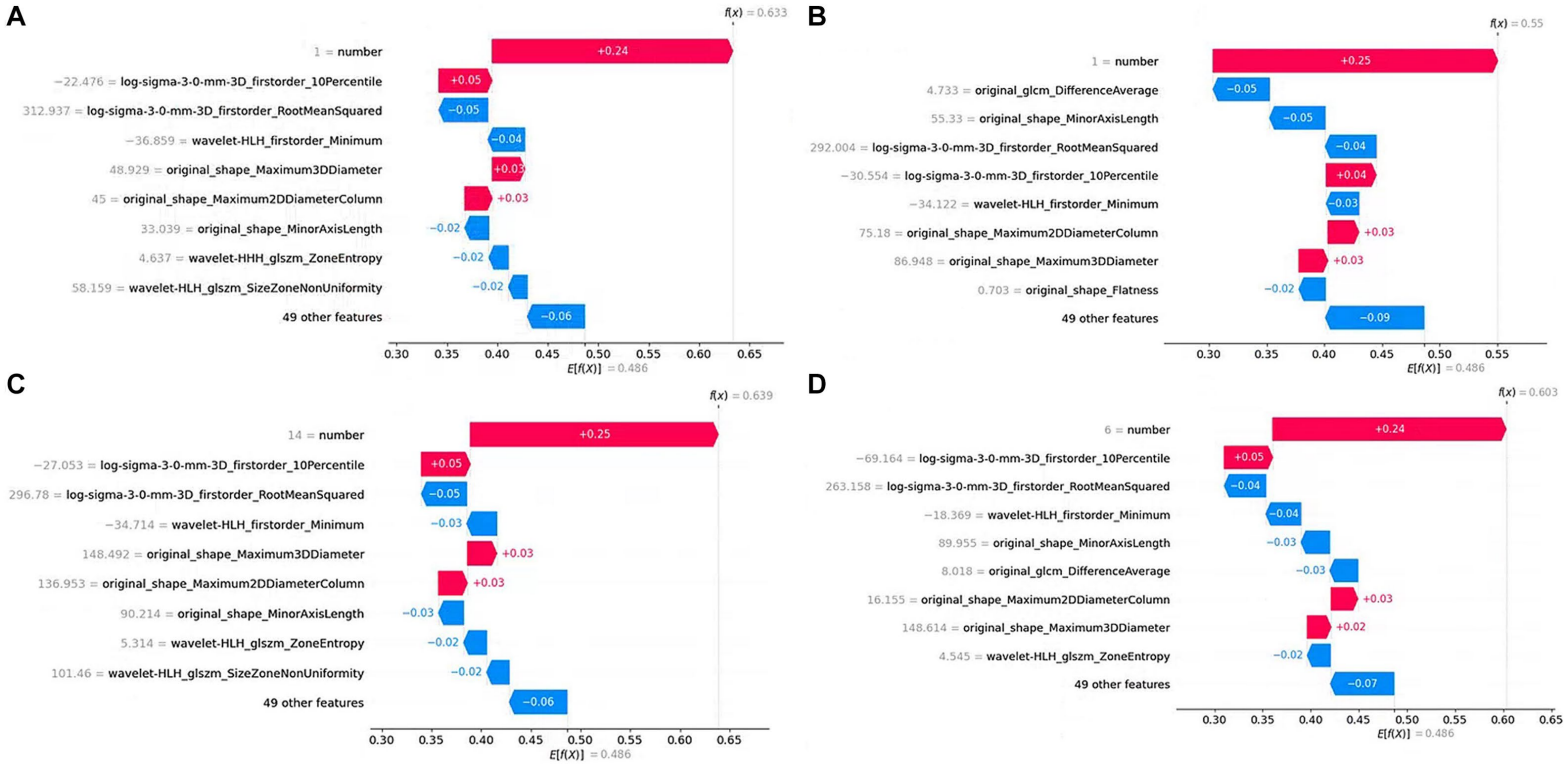
Waterfall plot of single sample prediction. The y-axis represents the feature name, the gray number next to the feature name is the feature value, and the number on the figure represents the feature contribution to the prediction result. **(A)**, **(B)**, **(C)**, and **(D)** are predictions for four samples, respectively.

Figure 10 shows the contribution of features to the predicted value in the prediction process of four samples in the form of a waterfall plot.

## 4. Discussion

In this study, an integrated gliomas and Mets CAD pipeline, including the improved automatic segmentation model and the radiomics-based classifier, was proposed. Moreover, an ensemble decision strategy based on prior knowledge strategy (EDPK) was introduced in the pipeline to improve the performance of the traditional radiomics-based method.

Radiomics is an emerging medical image analysis method that can convert images into quantitative data. The radiomics features extracted from brain MRI can capture information on heterogeneous details between tumor locations, which helps radiologists make a fast and correct decision on the tumor type. Previous relevant studies have shown the great significance of radiomics features in predicting tumor types (8, 9, 14–17, 47); however, those studies were mainly based on small datasets from a single-center application, which limited the CAD performance and multi-center applications. In addition, manual segmentation or semiautomatic methods for tumor segmentation limited the model’s robustness. Furthermore, it was a challenge to segment Mets lesions due to their size and shape (48). The comparison between prior works and our novel pipeline is shown in Table 5.

**Table 5:** Comparison between prior studies and our novel pipeline.

Author	PN	Modality	AS	FS	Feature	Best performance
Tateishi et al. (14)	127 (G:73 M:53) Single center	T1-CE, T2, ADC	No	n/a	Histogram and Texture	SVM AUC: 0.92
Dong et al. (15)	120 (G:60 M:60) Single center	T1, T1-CE T2	No	ComBat harmonization method	Shape, First-order, Texture,	Agreement of all five models ACC:0.94, AUC SEN:1, SPE:0.89
Liu et al. (16)	268 (G:140 M:128) Single center	T1-CE	No	Boruta selection	Shape, First-order, Texture, Wavelet transform, LoG	Random Forest ACC: 0.85 AUC: 0.97
Priya et al. (17)	120 (G:60 M:60) Single center	T1, T1-CE, T2, FLAIR, ADC	No	LASSO and elastic net	Shape, First-order, Texture	LASSO ACC:0.892 AUC:0.953
de Causans et al. (47)	180 (G:92 M:88) Single center	T1-CE	No	Yeo–Johnson scaling feature	Shape, First-order, Texture	LogReg ACC: 0.80 SEN:0.887 SPE:0.897
**Ours**	**1777 (G: 1002 M:755) Six centers**	T1-CE, T2-flair	No	Mann–Whitney U-test, LASSO+ MI, LASSO+RFE_RF	Shape, First-order, High-order Texture, Wavelet transform, LoG,**Lesion number**	**RSV model ACC:0.9144 AUC:0.9736 SEN:0.9144 SPE:0.9132**
Ours with EDPK			Yes			**RSV model with EDPK ACC:0.8950 AUC:0.9585 SEN:0.8961 SPE:0.8961**

G, gliomas; M, brain metastasis; n/a, not available; FS, feature selection method; PN, patient number; AS, automatic segmentation.

The bold values indicate the highest results.

Given the above issues, first, the effectiveness of several combinations of feature selection methods and classifiers was validated in the traditional radiomics pipeline and the importance of adding the clinical feature (lesion number) was demonstrated. Then, to interpret the results of the model by analyzing the importance of selected features, the bar chart and the SHAP model were used to visualize the importance of features. The top three radiomics features from the original MRI that contributed most to classifying gliomas and Mets were “shape-Flatness,” “shape-Maximum3D-Diameter,” and “shape-Sphericity” from the original image. “shape-Flatness” shows the relationship between the largest and smallest principal components in the ROI shape. “shape-Maximum3D-Diameter” feature is a measure of the largest pairwise Euclidean distance between tumor surface mesh vertices. “shape-Sphericity” calculates the ratio of the perimeter of the tumor region to the perimeter of a circle with the same surface area as the tumor region. The RSV model and LASSO selection achieved the best classification performance with an ACC of 0.9144 and AUC of 0.9736, based on the radiologist-delineated ROI.

Then, an integrative gliomas and Mets CAD method with automatic lesion segmentation and radiomics-based classification was developed. The diagnostic performance of our method was validated on the testing set and measured by accuracy, sensitivity, specificity, and AUC. For automatic brain tumor segmentation, two segmentation models for gliomas and Mets were trained separately due to the challenge of segmenting the Mets lesion. Both two models introduced the DenseNet (31) and Self-Attention (32) mechanisms based on the U-net (30) architecture. The Self-Attention mechanism was beneficial in capturing the internal correlation of features, solving the long-distance dependency problem, and improving the segmentation precision. The dense convolution was conducive to training deeper network structures and enhancing feature propagation and feature reuse. Finally, the glioma segmentation model yielded three segmented regions, and the Mets segmentation model yielded two segmented regions. For tumor segmentation, we have calculated dice coefficients, PPV, and sensitivity for segmentation in the whole tumor region, tumor core, and enhancing tumor region, as specified in Supplementary Table S12. Compared with the results of other state-of-the-art segmentation models, our two automatic segmentation models had higher dice coefficients (Table 3) on the testing data of gliomas and Mets. These regions were compatibly applied to all two MRI sequences (T1-CE and T2-flair). The ROI was the combination of these regions and was merged for subsequent quantitative feature extraction.

Moreover, an EDPK strategy was introduced in the pipeline to reduce the impact of automatic segmentation uncertainty on final classification performance and improve accuracy in the clinical diagnostic process. For each test case, two automatic segmentation results were obtained after post-processing and the EDPK strategy adaptively determined the ROI used to extract features and the weights in our RSV model based on the lesion number. In the current study, the EDPK strategy demonstrated better performance in differentiating gliomas and Mets with ACC of 0.8950, and AUC of 0.9585 in the testing set and could improve the classification performance of the RSV model under different lesion segmentation precision.

In brief, the input of our pipeline in the testing stage only requires two conventional brain MRI sequences (T1-CE, T2-flair), which even could be clinically implemented in resource-limited institutions. Moreover, it will give the tumor ROI segmentation results and the predicted tumor type diagnosed in real time.

## 5. Conclusion

In conclusion, radiomics methods have shown great potential in the field of brain tumor diagnosis, and the combinations of feature selection methods and classifiers were validated in the traditional radiomics pipeline. The proposed computer-aided method for diagnosing gliomas and Mets with automatic lesion segmentation and EDPK strategy improved the automatic lesion segmentation and diagnosis performance and even could be clinically implemented in resource-limited institutions. However, the current study collected only traditional MRI sequences and did not involve more sequences. Moreover, the ensemble decision strategy can only find a local optimal result. In future, combining multiple methods could solve the global optimum problem, by further expanding sample size, incorporating updated segmentation and classification methods in the pipeline to optimize the diagnostic method, and conducting more multi-center prospective studies.

## Data availability statement

The original contributions presented in the study are included in the article/Supplementary material, further inquiries can be directed to the corresponding authors.

## Ethics statement

Ethical review and approval was not required for the study on human participants in accordance with the local legislation and institutional requirements. Written informed consent from the participants was not required to participate in this study in accordance with the national legislation and the institutional requirements.

## Author contributions

LY and ZY contributed to the conception and design of the study, data analysis and interpretation, and manuscript writing. DG contributed to get the administrative support and provide the study materials and review of manuscript. LZ and LS contributed to the collection and assembly of data. All authors contributed to the article and approved the submitted manuscript.

## Funding

This study was supported by the National Natural Science Foundation of China (8237071280), “Clinical Medicine Research Pilot Project” of Shanghai Medical College of Fudan University DGF501022/015, Fudan University (gyy_yc_2020–8), Greater Bay Area Institute of Precision Medicine (Guangzhou), Fudan University (21618) and (KCH2310094), Shanghai Hospital Development Center (SHDC2020CR3020A), the Medical Engineering Joint Fund of Fudan University, Shanghai Municipal Commission of Science and Technology (22TS1400900, 23S31904100, 22ZR1409500), Shanghai Municipal Health Commission Project No.201940221, Shanghai Chest Hospital Project of Collaborative Innovation No. YJXT20190210Z, and Interdisciplinary program of Shanghai Jiaotong University No. YG2021QN123.

## Conflict of interest

The authors declare that the research was conducted in the absence of any commercial or financial relationships that could be construed as a potential conflict of interest.

## Publisher’s note

All claims expressed in this article are solely those of the authors and do not necessarily represent those of their affiliated organizations, or those of the publisher, the editors and the reviewers. Any product that may be evaluated in this article, or claim that may be made by its manufacturer, is not guaranteed or endorsed by the publisher.

## Footnotes

^1^ https://github.com/ANTsX/ANTsPy

^2^ https://scipy.org/

^3^ https://scikit-learn.org/stable/
