# Supplementary material for: A brain tumor computer-aided diagnosis method with automatic lesion segmentation and ensemble decision strategy
Source: Front Med (Lausanne). 2023 Sep 29;10:1232496. doi: 10.3389/fmed.2023.1232496 (PMC10576559; doi:10.3389/fmed.2023.1232496)
Supplement: Supplementary file 1 [file Table_1.DOCX]

Supplementary Material

# Supplementary Tables

**Table S1**. Distribution of patients in the training cohort and testing cohort

| Patient | Gliomas | Mets | Total |
| --- | --- | --- | --- |
| Training set | 738 | 540 | 1278 |
| Validation set | 184 | 135 | 319 |
| Testing set | 100 | 100 | 200 |
| Total | 1022 | 775 | 1797 |

**Table S2**. A summary of radiomics features extracted

14 shape features describing the three-dimensional size and shape of the ROI, which were independent of the gray level intensity distribution were extracted. From the original images and the transformed images (applying wavelet transform or Laplacian of Gaussian (LoG) filtering), 198 First-order features describing the distribution of the intensities were extracted, while 748 High-order texture features were computed to describe the patterns, or the high-order intensity distributions with four methods, including gray-level co-occurrence matrix (GLCM), gray-level run length matrix (GLRLM), gray level size zone matrix (GLSZM), gray level dependence matrix (GLDM). Totally, 960 features were extracted from each MR sequences.

| **Feature Classes** | | **Feature Names** |
| --- | --- | --- |
| Shape Features | | Elongation, Flatness, Least Axis Length, Major Axis Length, Maximum 2D Diameter Column, Maximum 2D Diameter Row, Maximum 2D Diameter Slice, Maximum 3D Diameter, Mesh Volume, Minor Axis Length, Sphericity, Surface Area, Surface Volume Ratio, Voxel Volume |
| First-order Features | | Maximum, Median, Minimum, Mean, Energy, Entropy, Variance, Kurtosis, Root Mean Square, Skewness, 10th Percentile, 90th Percentile, Mean Absolute Deviation, Uniformity, Range, Robust Mean Absolute Deviation, Total Energy, Interquartile Range |
| High-order texture  Features | GLCM  Features  (22) | Contrast, Correlation, Autocorrelation, Cluster Tendency, Sum Average, Sum Entropy, Sum Squares, Difference Average, Difference Variance, Difference Entropy, Cluster Prominence, Cluster Shade, Maximum Probability, Inverse Difference Moment, Informational Measure of Correlation 1/2, Inverse Difference Moment Normalized, Inverse Difference Normalized, Inverse Difference, Inverse Variance, Maximal Correlation Coefficient, Joint Average, Joint Energy, Joint Entropy |
|  | GLDM  Features  (14) | Dependence Entropy, Dependence Non-Uniformity, Dependence Non-Uniformity Normalized, Dependence Variance, Gray-Level Non-Uniformity, Gray-Level Variance, High Gray-Level Emphasis, Large Dependence Emphasis, Large Dependence High Gray-Level Emphasis, Large Dependence Low Gray-Level Emphasis, Low Gray-Level Emphasis, Small Dependence Emphasis, Small Dependence High Gray-Level Emphasis, Small Dependence Low Gray-Level Emphasis |
|  | GLRLM  Features  (16) | Gray-Level Non-uniformity, Gray-Level Non-uniformity Normalized, Gray-Level Variance, High Gray-Level Run Emphasis, Long Run Emphasis, Long Run High Gray-Level Emphasis, Long Run Low Gray-Level Emphasis, Low Gray-Level Run Emphasis, Run Entropy, Run Length Non-Uniformity, Run Length Non-Uniformity Normalized, Run Percentage, Run Variance, Short Run Emphasis, Short Run High Gray-Level Emphasis, Short Run Low Gray-Level Emphasis |
|  | GLSZM  Features  (16) | Gray-Level Non-Uniformity, Gray-Level Non-Uniformity Normalized, Gray-Level Non-Uniformity Normalized, High Gray-Level Zone Emphasis, Large Area Emphasis, Large Area High Gray-Level Emphasis, Large Area Low Gray-Level Emphasis, Low Gray-Level Zone Emphasis, Size Zone Non-Uniformity, Size Zone Non-Uniformity Normalized, Small Area Emphasis, Small Area High Gray-Level Emphasis, Small Area Low Gray-Level Emphasis, Zone Entropy, Zone Percentage, Zone Variance |

**Table S3**. The number of radiomics features extracted from different image types.

| Image Type | Shape Feature | First-Order Feature | High-Order Texture Feature | Total |
| --- | --- | --- | --- | --- |
| Original images | 14 | 18 | 68 | 100 |
| LoG images | 0 | 36 | 136 | 172 |
| Wavelet images | 0 | 144 | 544 | 688 |
| Total | 14 | 198 | 748 | 960 |

**Table S4.** Hyperparameter values of each model used in the evaluation.

| Model | Hyperparameter values |
| --- | --- |
| GNB | priors=None, var_smoothing=1e-09 |
| LR | penalty=’l1’, solver=’liblinear’, max_iter=1000 |
| RF | n_estimators=74 |
| SVM-Poly | kernel=’poly’, C=50000.0, class_weight=’balanced’, gamma=0.001, probability=True |
| SVM-RBF | kernel=’rbf’, C =1024, gamma = 0.0006, probability=True |
| XGBoost | n_estimators=28, objective=’binary: hinge’, use_label_encoder=False |

**Table S5**. The weighted macro averaged performance of 2-class brain tumor classification under different ML classifiers according to 58 features

| **Features** | **Model** | **PRE** | **Recall** | **F1-score** | **SEN** | **SPE** |
| --- | --- | --- | --- | --- | --- | --- |
| 57 selected features by LASSO + NL | LR（L1） | 0.9065  (0.9260,0.8881) | 0.9055  (0.8756,0.9338) | 0.9054  (0.9001,0.9104) | 0.9055  (0.8756,0.9338) | 0.9038  (0.9338,0.8756) |
|  | GNB | 0.8247  (0.8030,0.8454) | 0.8237  (0.8446,0.8039) | 0.8237  (0.8232,0.8241) | 0.8237  (0.8446,0.8039) | 0.8248  (0.8039,0.8446) |
|  | SVM (Poly) | 0.9025  (0.9185,0.8873) | 0.9018  (0.8756,0.9265) | 0.9017  (0.8966,0.9065) | 0.9018  (0.8756,0.9265) | 0.9003  (0.9265,0.8756) |
|  | SVM  (rbf) | 0.9067  (0.9379,0.8773) | 0.9043  (0.8601,0.9461) | 0.9040  (0.8973,0.9104) | 0.9043  (0.8601,0.9461) | 0.9019  (0.9461,0.8601) |
|  | **RF** | **0.9139**  (0.9319,0.8970) | **0.9131**  (0.8860,0.9387) | **0.9130**  (0.9084,0.9174) | **0.9131**  (0.8860,0.9387) | **0.9174**  (0.9387,0.8860 |
|  | XGBoost | 0.8944  (0.9016,0.8876) | 0.8942  (0.8782,0.9093) | 0.8942  (0.8898,0.8983) | 0.8942  (0.8782,0.9093) | 0.8933  (0.9093,0.8782) |

**Table S6.** The ACC and average-AUC under different ML classifiers according to 58 features.

| **Features** | **Model** | **ACC** | **AUC** |
| --- | --- | --- | --- |
| 57 features selected by lasso + NL | LR(L1) | 0.9055 | 0.8693 |
|  | GNB | 0.8237 | 0.9035 |
|  | SVM (poly) | 0.9018 | 0.9582 |
|  | SVM (rbf) | 0.9043 | 0.9654 |
|  | **RF** | **0.9131** | **0.9705** |
|  | XGBoost | 0.8942 | 0.8870 |

**Table S7.** The weighted macro averaged performance of 2-class brain tumor classification under different ML classifiers according to 30 features selected after MI method

| **Features** | **Model** | **PRE** | **Recall** | **F1-score** | **SEN** | **SPE** |
| --- | --- | --- | --- | --- | --- | --- |
| 30 features selected by lasso and MI | LR(L1) | 0.8929 (0.9144,0.8727) | 0.8917  (0.8575,0.9240) | 0.8915  (0.8850,0.8976) | 0.8917  (0.8575,0.9240) | 0.8  (0.9240,0.8575) |
|  | GNB | 0.8200  (0.8240,0.8162) | 0.8199  (0.8005,0.8382) | 0.8198  (0.8121,0.8271) | 0.8199  (0.8005,0.8382) | 0.8188  (0.8382,0.8005) |
|  | SVM  (poly) | 0.8966  (0.9174,0.8770) | 0.8955  (0.8627,0.9265) | 0.8953  (0.8892,0.9011) | 0.8955  (0.8627,0.9265) | 0.8937  (0.9265,0.8627) |
|  | SVM  (rbf) | 0.9048  (0.9189,0.8915) | 0.9043  (0.8808,0.9265) | 0.9042  (0.89950.9087) | 0.9043  (0.8808,0.9265) | 0.9030  (0.9265,0.8808) |
|  | RF | 0.9058  (0.9147, 0.8974) | 0.9055  (0.8886,0.9216) | 0.9055  (0.9014,0.9093) | 0.9055  (0.8886,0.9216) | 0.9046  (0.9216,0.8886) |
|  | XGBoost | 0.8920  (0.9011,0.8833) | 0.8917  (0.8731,0.9093) | 0.8916  (0.8868,0.8961) | 0.8917  (0.8731,0.9093) | 0.8907  (0.9093,0.8731) |

**Table S8.** The ACC and average-AUC under different ML classifier according to 30 features selected after MI method

| **Features** | **Model** | **ACC** | **AUC** |
| --- | --- | --- | --- |
| 30 features selected by lasso and MI | LR(L1) | 0.8917 | 0.9609 (0.9609, 0.9609) |
|  | GNB | 0.8174 | 0.8967 (0.8947,0.8990) |
|  | SVM (poly) | 0.8955 | 0.9493 (0.9493, 0.9493) |
|  | **SVM (rbf)** | **0.9043** | **0.9671** (0.9671,0.9671) |
|  | RF | 0.8992 | 0.9656 (0.9656, 0.9656) |
|  | XGBoost | 0.8917 | 0.8968 (0.8968,0.8968) |

The better-performing model for features selected by LASSO and MI was SVM using the RBF kernel function, with an AUC of 0.9671, an accuracy of 0.9043, a sensitivity of 0.9043, a specificity of 0.9030, a precision of 0.9048, a recall of 0.9043, and an F1-score of 0.9043, and RF with an AUC of 0.9656, an accuracy of 0.8992, a sensitivity of 0.9055, a specificity of 0.9046, a precision of 0.9058, a recall of 0.9055, and an F1-score of 0.9055.

**Table S9.** The weighted macro averaged performance of 2-class brain tumor classification under different ML classifier according to 30 features selected after REF-RF method

| **Features** | | **Model** | **PRE** | **Recall** | **F1-score** | **SEN** | **SPE** |
| --- | --- | --- | --- | --- | --- | --- | --- |
| 30 features selected by lasso and RFE-RF | LR(L1) | | 0.8895  (0.9136,0.8667) | 0.8879  (0.8497,0.9240) | 0.8877  (0.8805,0.8944) | 0.8879  (0.8497,0.9240) | 0.8858  (0.9240,0.8497) |
|  | GNB | | 0.8463  (0.8743,0.8198) | 0.8438  (0.7927,0.8922) | 0.8433  (0.8315,0.8545) | 0.8438  (0.7927,0.8922) | 0.8411  (0.8922,0.7927) |
|  | SVM  (poly) | | 0.9068  (0.9084,0.9053) | 0.9068  (0.8990,0.9142) | 0.9068  (0.9036,0.9098) | 0.9068  (0.8990,0.9142) | 0.9063  (0.9142,0.8990) |
|  | SVM  (rbf) | | 0.9019  (0.9074,0.8966) | 0.9018  (0.8886,0.9142) | 0.9017  (0.8979,0.9053) | 0.9018  (0.8886,0.9142) | 0.9010  (0.9142,0.8886) |
|  | **RF** | | **0.9126**  (0.9293,0.8967) | **0.9118**  (0.8860,0.9363) | **0.9117**  (0.9072,0.9161) | **0.9118**  (0.8860,0.9363) | **0.9105**  (0.9363,0.8860) |
|  | XGBoost | | 0.9095  (0.9176,0.9019) | 0.9093  (0.8938,0.9240) | 0.9093  (0.9055,0.9128) | 0.9093  (0.8938,0.9240) | 0.9085  (0.9240,0.8938) |

**Table S10.** The ACC and AUC under different ML classifier according to 30 features selected after RFE-RF method

| **Features** | **Model** | **ACC** | **AUC** |
| --- | --- | --- | --- |
| 30 features selected by lasso and RFE-RF | LR(L1) | 0.8879 | 0.9565 |
|  | GNB | 0.8438 | 0.9010 |
|  | SVM (Poly) | 0.9068 | 0.9597 |
|  | SVM (RBF) | 0.9018 | 0.9688 |
|  | **RF** | **0.9118** | **0.9713** |
|  | XGBoost | 0.9093 | 0.9089 |

The best-performing model for features selected by LASSO and RFE-RF was Random Forest, with an AUC of 0.9713, an accuracy of 0.9118, a sensitivity of 0.9118, a specificity of 0.9105, a precision of 0.9126, a recall of 0.9118, and an F1-score of 0.9117 for the classifier.

**Table S11.** The result of the Delong test between our model and the other classifier.

| Compared model | 95% AUC CI | p-value |
| --- | --- | --- |
| Our VS LR(L1) | [0.9641844, 0.98297348] | 0.001 |
| Our VS GNB | [0.9641844, 0.98297348] | 0.000 |
| Our VS SVM (poly) | [0.9641844, 0.98297348] | 0.004 |
| Our VS XGBoost | [0.9641844, 0.98297348] | 0.0001 |

**Table S12.** The segmentation result of the whole tumor region, tumor core, and enhancing tumor region

| Tumor type | Segmented Region | Dice | PPV | Sensitivity |
| --- | --- | --- | --- | --- |
| Brain metastases | WT | 0.8464 | 0.8636 | 0.8425 |
|  | TC | 0.7810 | 0.8172 | 0.7633 |
|  | ET | 0.7813 | 0.8178 | 0.7632 |
| Gliomas | WT | 0.8451 | 0.8904 | 0.8178 |
|  | TC | 0.5757 | 0.8596 | 0.5121 |
|  | ET | 0.1128 | 0.2612 | 0.1971 |

Note:

WT: the whole tumor region

TC: the tumor core

ET: the enhancing tumor

PPV: Positive Predictive Value

Abbreviations are as follow:

NL: the lesion number

MI: Mutual Information

RFE_RF: Recursive Feature Elimination random forest

LR(L1): Logistic Regression with L1 penalty

GNB: Gaussian Naïve Bayes

RF: Random Forest

SVM(Poly): Support Vector Machine with Poly kernel

SVM(RBF): Support Vector Machine with RBF function kernel

XGBoost: Extreme Gradient Tree

ACC: accuracy

AUC: area under the receiver operating characteristic curve

PRE: precision

SEN: sensitivity

SPE: specificity
