# Supplementary figures and images for: A brain tumor computer-aided diagnosis method with automatic lesion segmentation and ensemble decision strategy
Source: Front Med (Lausanne). 2023 Sep 29;10:1232496. doi: 10.3389/fmed.2023.1232496 (PMC10576559; doi:10.3389/fmed.2023.1232496)

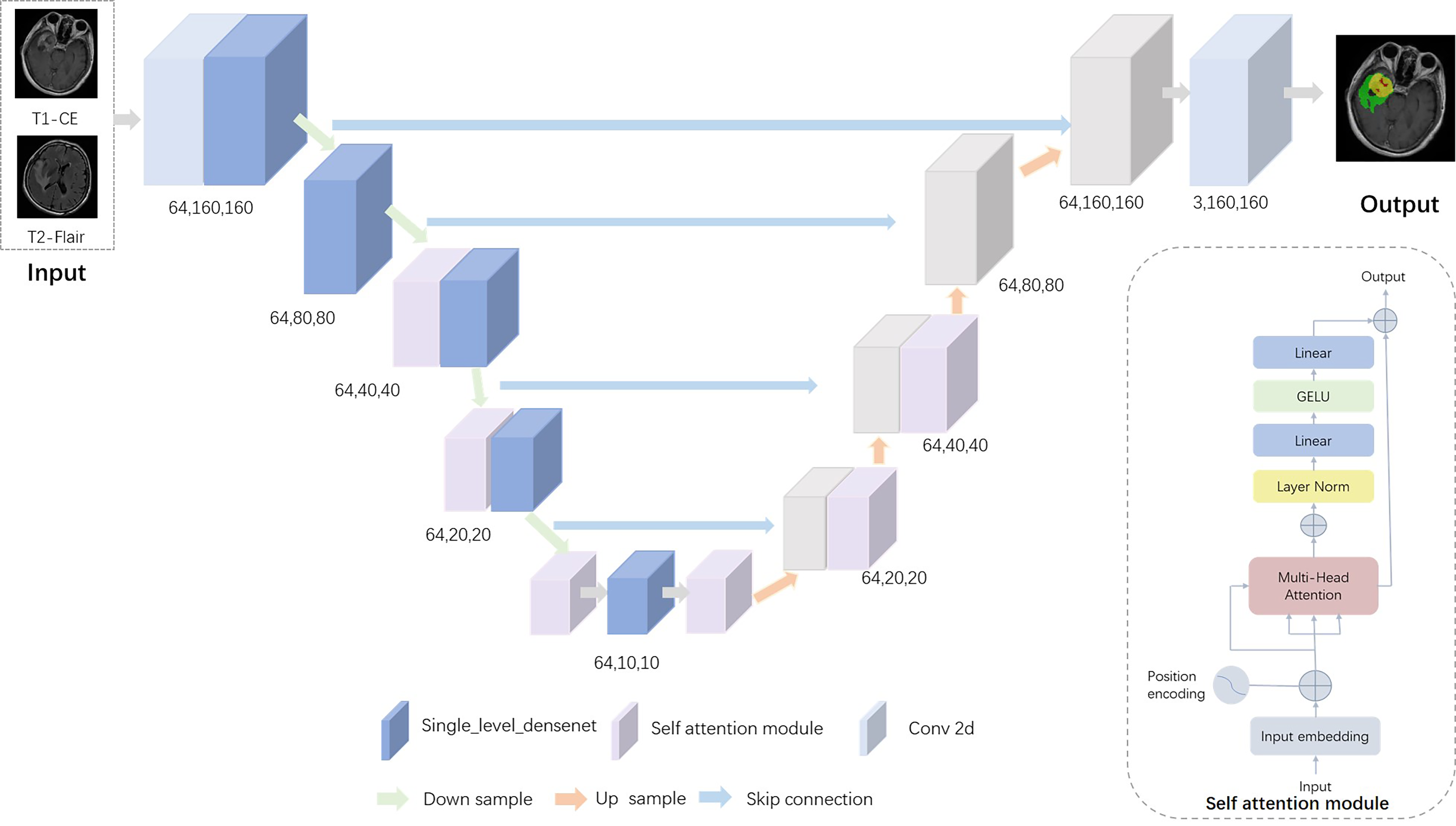

Supplement: Supplementary file 2 [file Image_1.TIF]

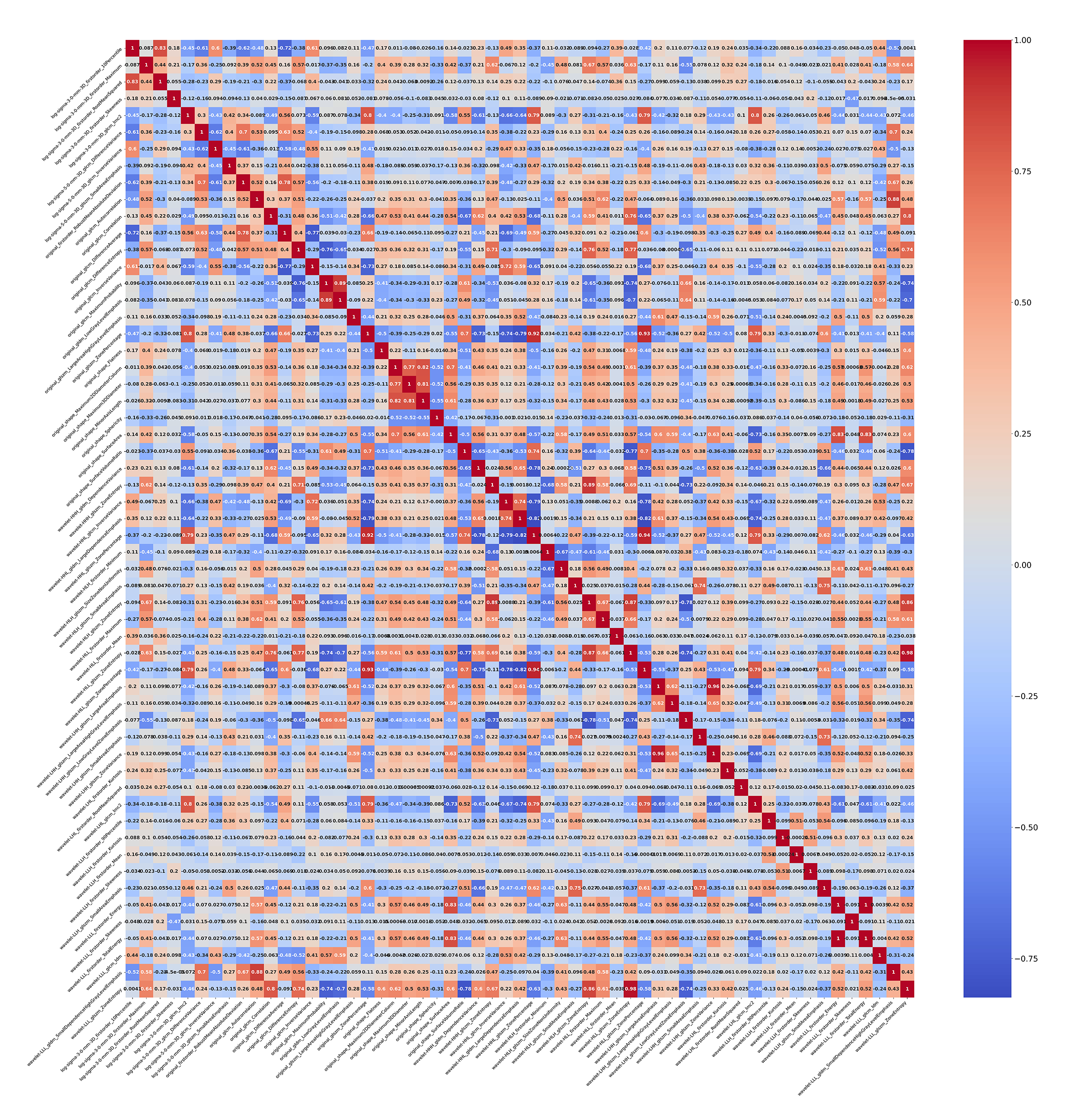

Supplement: Supplementary file 3 [file Image_2.TIF]

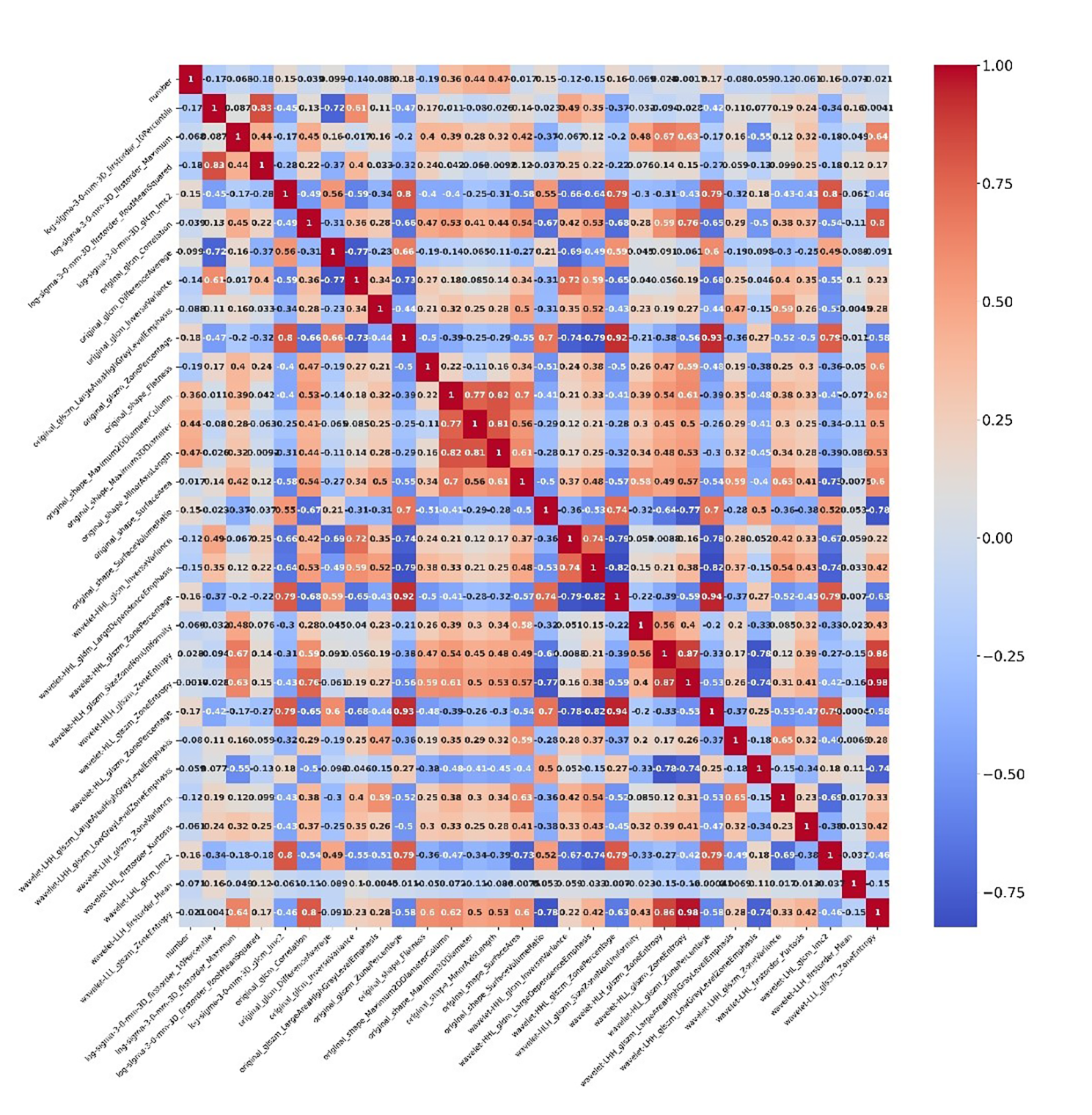

Supplement: Supplementary file 4 [file Image_3.TIF]

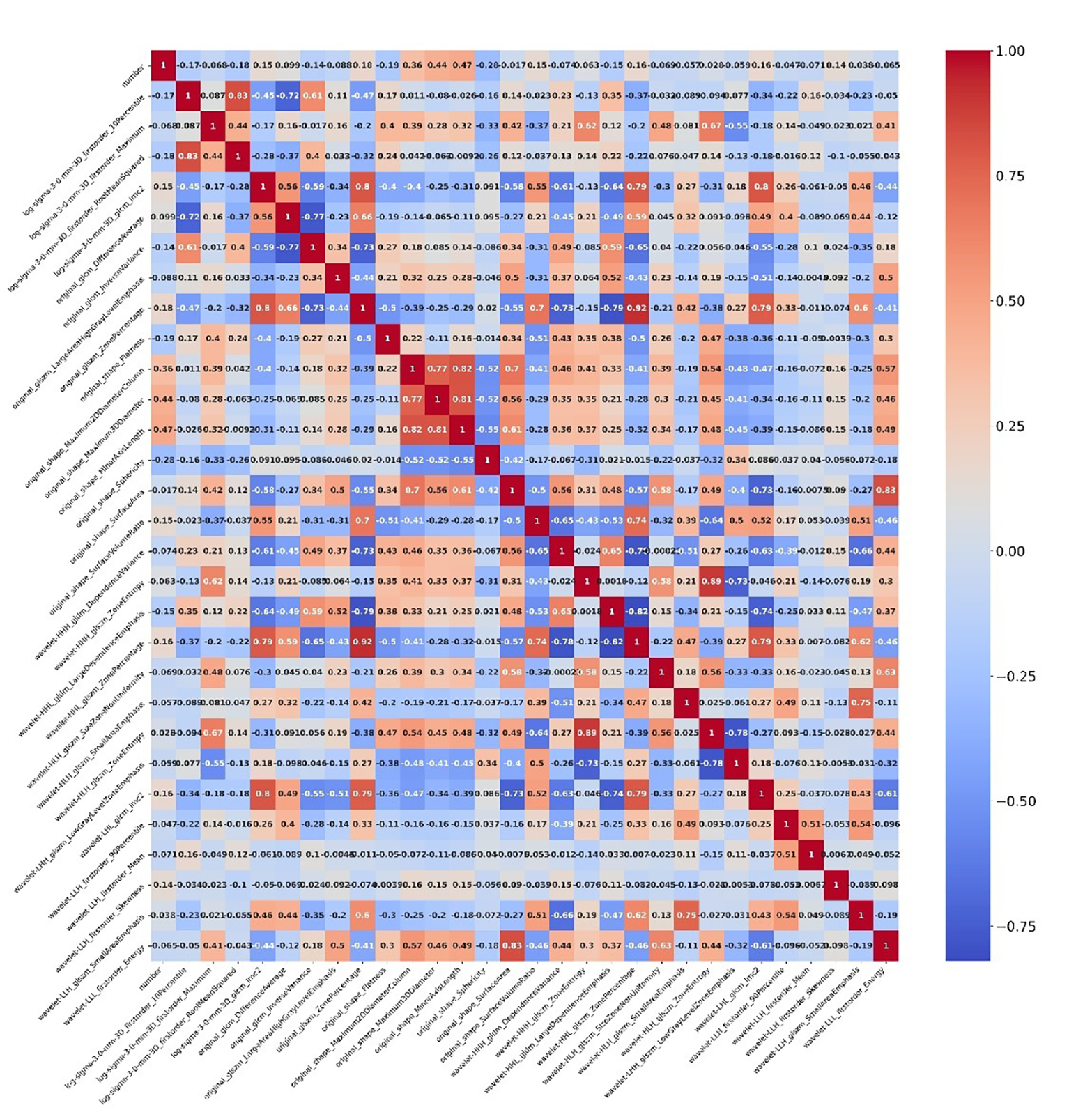

Supplement: Supplementary file 5 [file Image_4.TIF]

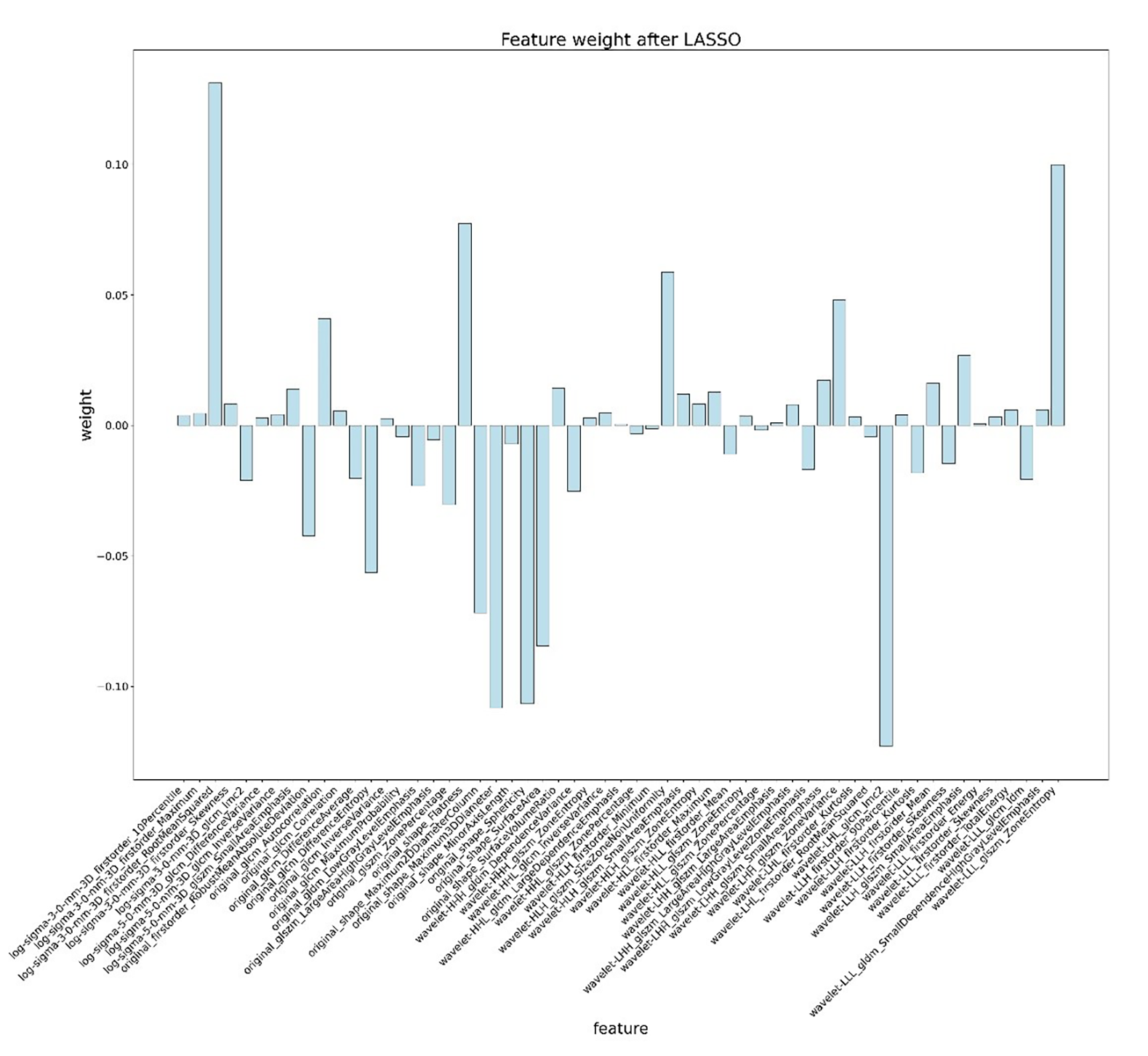

Supplement: Supplementary file 6 [file Image_5.TIF]

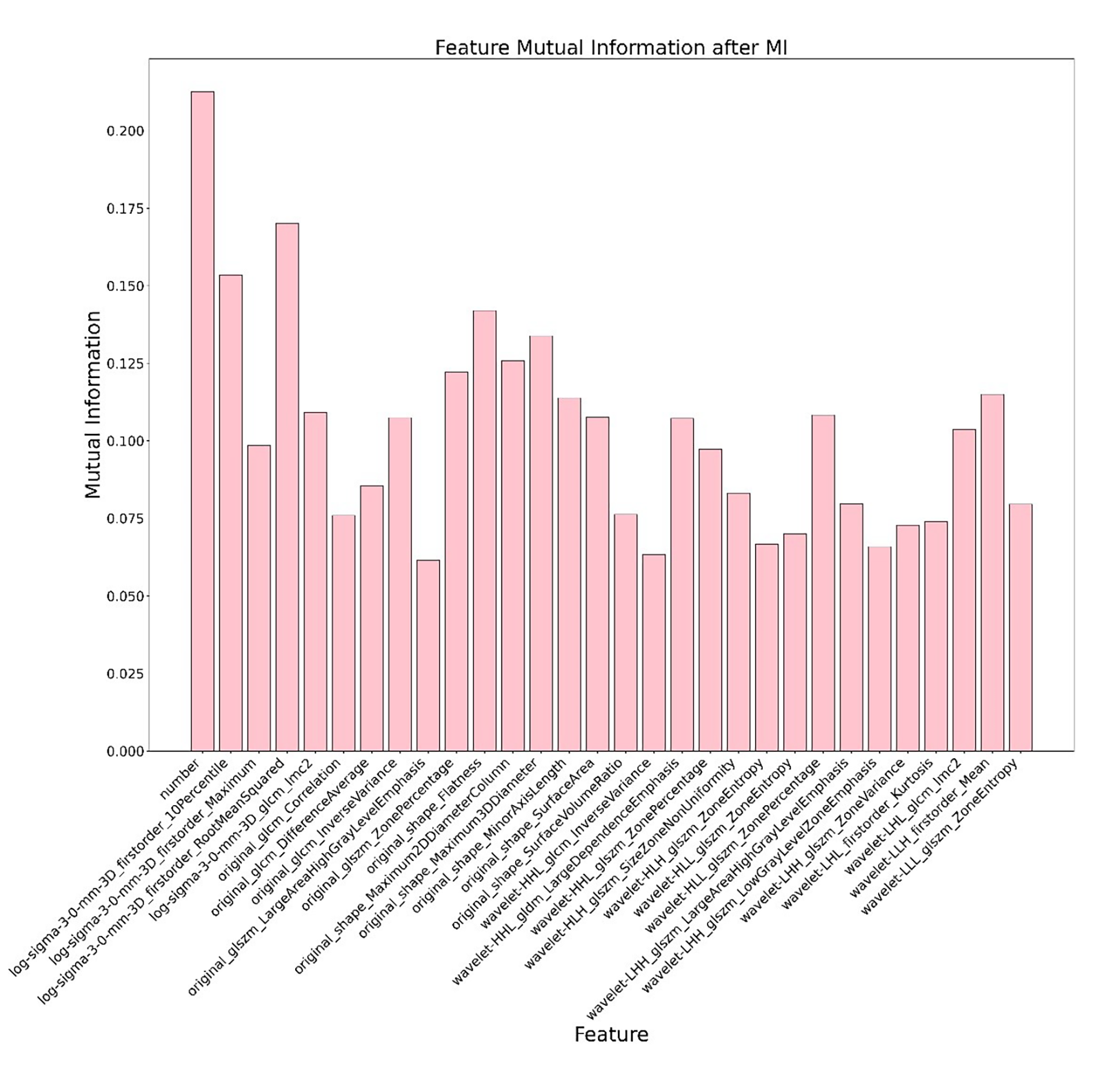

Supplement: Supplementary file 7 [file Image_6.TIF]

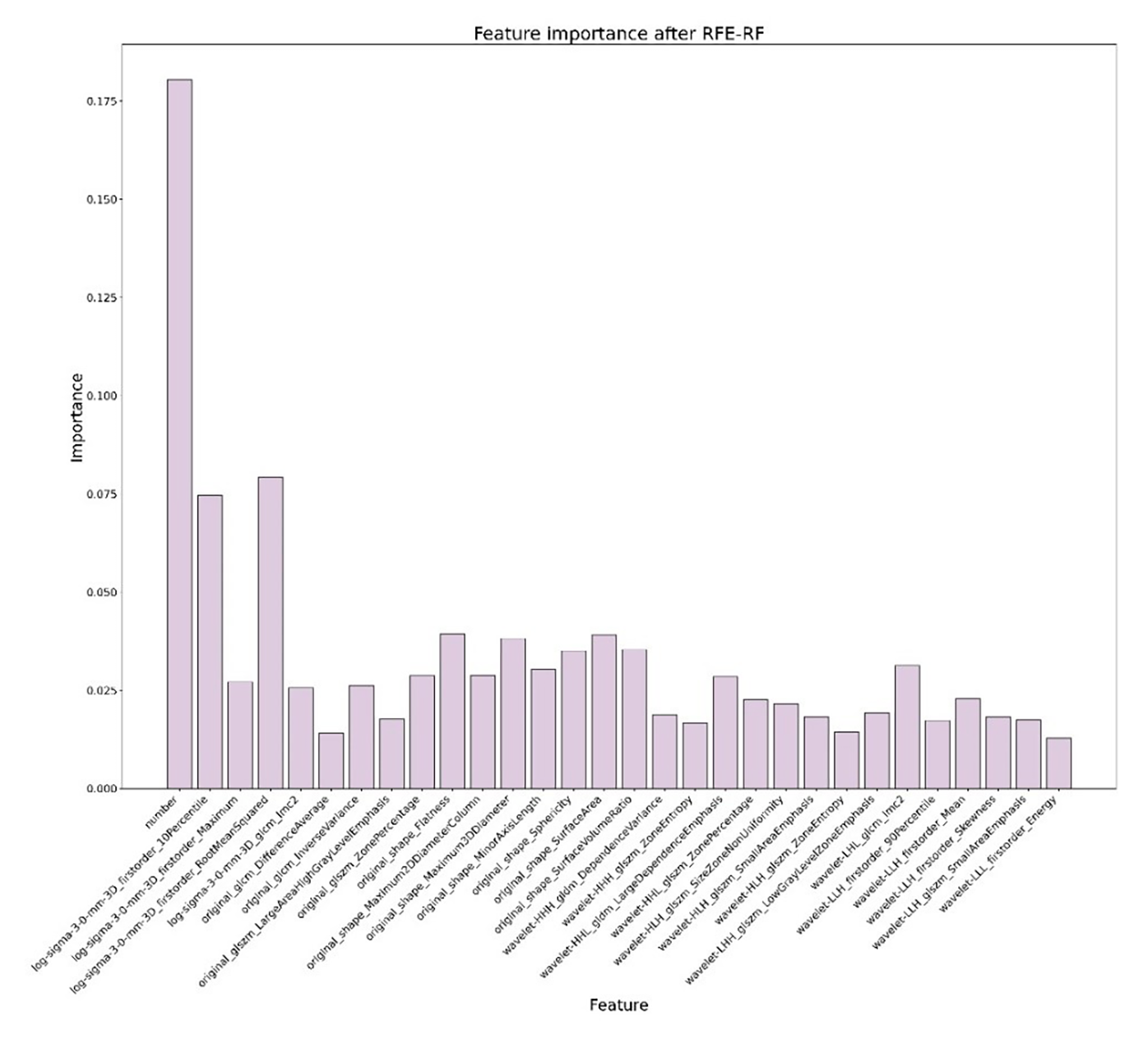

Supplement: Supplementary file 8 [file Image_7.TIF]

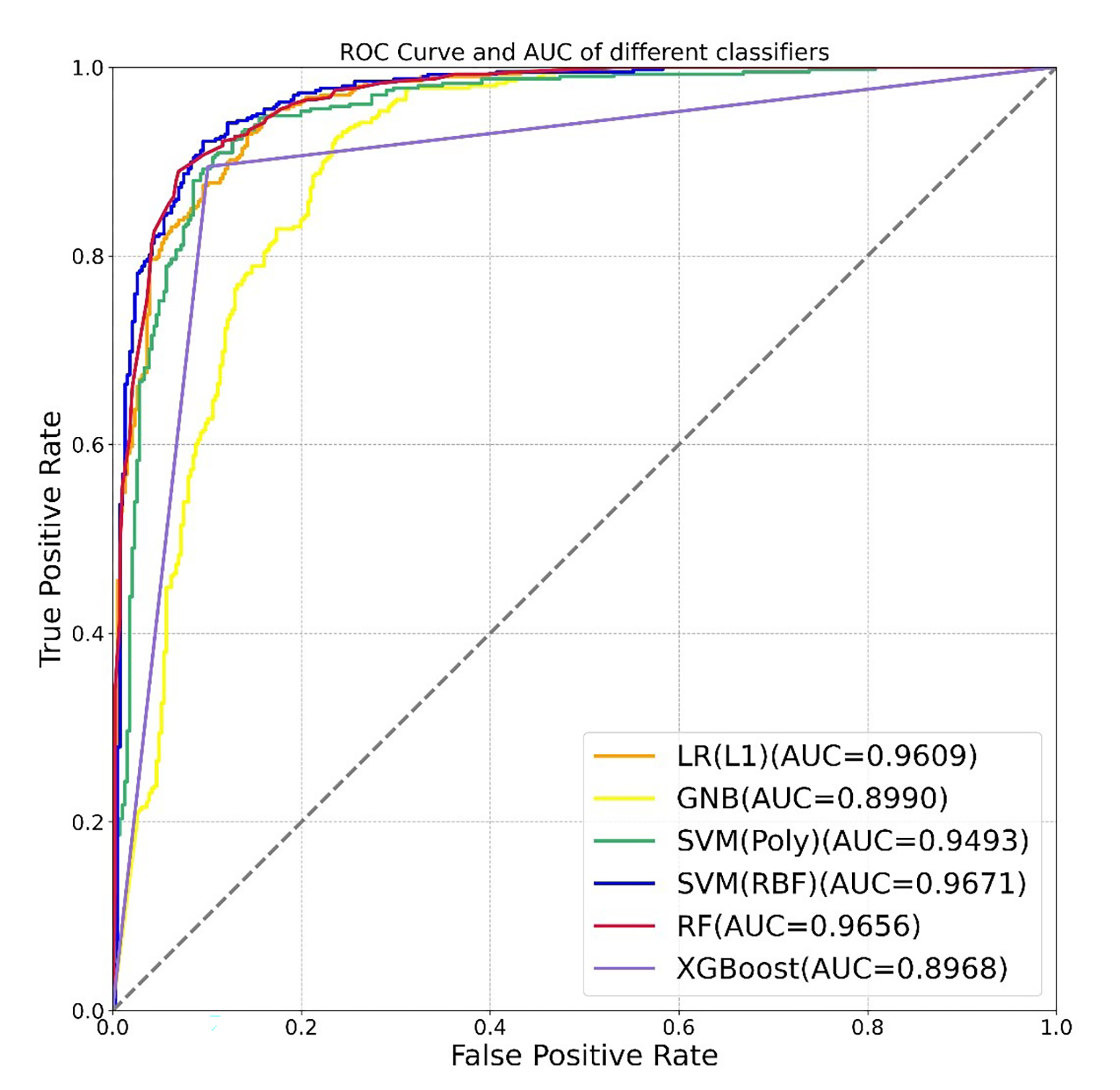

Supplement: Supplementary file 9 [file Image_8.TIF]

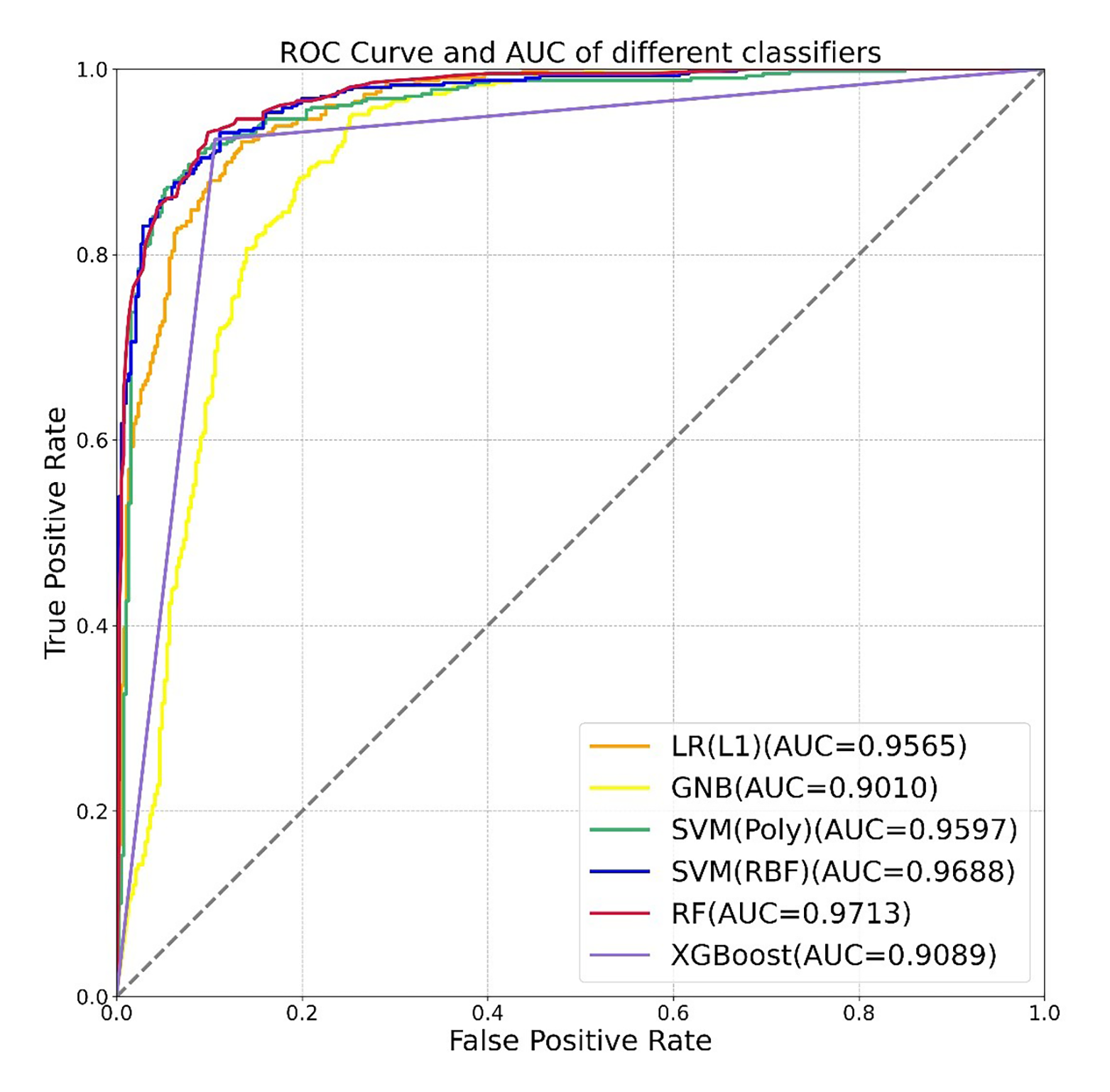

Supplement: Supplementary file 10 [file Image_9.TIF]
